# An Evaluation of the COVID-19 Pandemic and Perceived Social Distancing Policies in Relation to Planning, Selecting, and Preparing Healthy Meals: An Observational Study in 38 Countries Worldwide

**DOI:** 10.3389/fnut.2020.621726

**Published:** 2021-02-04

**Authors:** Charlotte De Backer, Lauranna Teunissen, Isabelle Cuykx, Paulien Decorte, Sara Pabian, Sarah Gerritsen, Christophe Matthys, Haleama Al Sabbah, Kathleen Van Royen, the Corona Cooking Survey Study Group

**Affiliations:** ^1^Department of Communication Sciences, Faculty of Social Sciences, University of Antwerp, Antwerp, Belgium; ^2^Tilburg Center for Cognition and Communication, Tilburg School of Humanities and Digital Sciences, Tilburg University, Tilburg, Netherlands; ^3^School of Population Health, University of Auckland, Auckland, New Zealand; ^4^Clinical and Experimental Endocrinology, KU Leuven, Leuven, Belgium; ^5^Public Health Nutrition Department, Zayed University, Dubai, United Arab Emirates; ^6^Family Medicine and Population Health, Faculty of Medicine and Health Sciences, University of Antwerp, Antwerp, Belgium

**Keywords:** food literacy, food planning, food preparation, food selection, nutrition, COVID-19, psychological distress, time availability

## Abstract

**Objectives:**

To examine changes in planning, selecting, and preparing healthy foods in relation to personal factors (time, money, stress) and social distancing policies during the COVID-19 crisis.

**Methods:**

Using cross-sectional online surveys collected in 38 countries worldwide in April-June 2020 (*N* = 37,207, *M*age 36.7 *SD* 14.43, 73.6% women), we compared changes in food literacy behaviors to changes in personal factors and social distancing policies, using hierarchical multiple regression analyses controlling for sociodemographic variables.

**Results:**

Increases in planning (4.7 *SD* 1.2, 4.9 *SD* 1.3), selecting (3.8 *SD* 1.7, 3.8 *SD* 1.7), and preparing (4.6 *SD* 1.3, 4.7 *SD* 1.3) healthy foods were found for women and men, and positively related to perceived time availability among women and stay-at-home policies for planning and preparing in women. Psychological distress was a barrier for women, and an enabler for men. COVID-19 induced financial stress was a barrier depending on various sociodemographic variables (all *p* < 0.01).

**Conclusion:**

Stay-at-home policies and feelings of having more time during COVID-19 seem to have improved food literacy among women. Stress and other social distancing policies relate to food literacy in more complex ways, highlighting the necessity of a health equity lens.

## Introduction

At the onset of the global COVID-19 crisis, “panic buying” of grocery staples and time-intensive food preparation activities emerged worldwide (1, 2). The crisis and social distancing policies created unique situations worldwide that allow us to study people and their circumstances in relation to food and health, which is needed for future intervention approaches (3). The goal of this study is to evaluate people's experience of and responses to the COVID-19 crisis and social distancing policies in relation to three behavioral food literacy components: planning, selecting, and preparing healthier foods (4, 5) that have a direct impact on of the individual and household? (6).

A lack of time, financial struggles, and stress are well-known personal barriers to food literacy (7–10). Since the onset of the COVID-19 crisis, many people around the world have experienced (partial) unemployment, and experts anticipate long-term economic consequences that will also affect health (11). We hypothesize that due to the COVID-19 crisis, financial stress and, if applicable, loss of income will have had negative associations with food literacy behavior.

The COVID-19 crisis has also distorted many peoples' perception of time (12). Policies to stay home may have given people the perception of having more time than usual, to the degree that initial findings concerning time and COVID-19 even mention boredom (13). The perception of having more hours in the day might also relieve people from the time-related stress of busy schedules, which is known to impede food literacy behaviors (14). We hypothesize that personal perceptions of having more time and contextual factors of being forced to stay home will have related positively to changes in food literacy behavior.

Next, the COVID-19 crisis and social distancing policies have caused considerable social and psychological distress (11). Psychological distress is known to have negative effects on nutrition behaviors (15). Eating behaviors are also part of food literacy behavior (4, 5), and negative effects of COVID-19-induced stress on planning, selecting, and preparing foods can also be expected. However, preparing foods (e.g., baking) potentially functions as a creative activity to relieve stress (16). Psychological distress caused by the COVID-19 crisis may thus relate to food literacy behavior in both positive and negative ways.

Based on these hypotheses, this study aims to investigate how the onset of the COVID-19 crisis and ensuing social distancing policies have influenced individual feelings that ultimately led to changes in planning, selecting, and preparing healthier foods in 38 countries worldwide. Acknowledging social inequities based on gender, age, educational attainment, employment status, income (for food), and the number of (adult) members in the household (3–5, 7, 10, 17), these factors will also be considered in this study as covariates.

## Materials and Methods

### Study Design and Setting

A cross-sectional online survey was launched in 38 countries worldwide^1^, using almost all native languages (all details are listed in Table 1) between April 17th and June 25th 2020. The survey consisted of multiple information blocks, of which only a few variables are used and reported in this paper. A full overview of the study protocol and survey is accessible via https://osf.io/nz9xf/files/.

**Table 1:** Detailed descriptive statistics (Means, Standard Deviations, and Valid Percentages) for the entire sample, weighted^a^ and subsamples of women and men, used in all analyses.

		**Total sample *N* = 37,207**		**Weighted sample used in analyses**	**Weighted female subsample**	**Weighted male subsample**	
	**Answer option**	***M* (*SD*) or *n* (valid %)**	**Missing values *n***	***M* (*SD*) or valid %**	***M* (*SD*) or valid %**	***M* (*SD*) or valid %**	**Significance of sex. differences based on *t*-tests (*M*, *SD*) or Chi-square (%)**
**Food literacy scores**							
Plan before COVID-19	1–7 Likert	4.70 (1.26)	0	4.66 (1.24)	4.77 (1.21)	4.36 (1.28)	*t*_16,156.28)_ = 28.33, *p* < 0.001
Plan during COVID-19	1–7 Likert	4.89 (1.34)	0	4.87 (1.31)	5.00 (1.27)	4.51 (1.36)	*t*_15,928.77)_ = 31.47, *p* < 0.001
Select before COVID-19	1–7 Likert	3.61 (1.65)	0	3.75 (1.66)	3.84 (1.66)	3.53 (1.66)	*t*_36,654)_ = 16.40, *p* < 0.001
Select during COVID-19	1–7 Likert	3.66 (1.71)	0	3.80 (1.71)	3.86 (1.71)	3.62 (1.68)	*t*_36,654)_ = 12.32, *p* < 0.001
Prepare food before COVID-19	1–7 Likert	4.60 (1.24)	0	4.56 (1.25)	4.69 (1.20)	4.22 (1.33)	*t*_15,486.65)_ = 30.64, *p* < 0.001
Prepare food during COVID-19	1–7 Likert	4.72 (1.29)	0	4.71 (1.29)	4.85 (1.23)	4.31 (1.38)	*t*_15,471.12)_ = 34.08, *p* < 0.001
**COVID-19 induced feelings**							
Financial stress	1–7 Likert	2.85 (1.76)	0	2.88 (1.74)	2.85 (1.73)	2.97 (1.78)	*t*_16,581.94)_ = −5.234, *p* < 0.001
Feel they have more time	1–7 Likert	4.18 (1.74)	0	4.15 (1.75)	4.15 (1.75)	4.17 (1.74)	*t*_36,654)_ = −1.183, *p* = 0.237
KESSLER 6	1–7 Likert	3.06 (1.28)	0	3.07 (1.26)	3.15 (1.25)	2.86 (1.26)	*t*_36,654)_ = 20.52, *p* < 0.001
**COVID-19 contextual factors**
Forced to work/stay home	Yes/No	29,558 (79.4%)	0	80.5%	82.00%	76.2%	$X_{\left(1\right)}^{2}$ = 154.74, *p* < 0.001
Public gatherings restricted	Yes/No	9,464 (25.4%)	0	27.1%	25.9%	30.5%	$X_{\left(1\right)}^{2}$ = 78.90, *p* < 0.001
Private gatherings restricted	Yes/No	5,508 (14.8%)	0	14.9%	14.3%	16.4%	$X_{\left(1\right)}^{2}$ = 25.75, *p* < 0.001
Restaurants closed	Yes/No	28,309 (76.1%)	0	77.4%%	79.1%	72.7%	$X_{\left(1\right)}^{2}$ = 168.79, *p* < 0.001
Bars/pubs closed	Yes/No	29,259 (78.6%)	0	79.7%	80.2%	78.3%	$X_{\left(1\right)}^{2}$ = 16.08, *p* < 0.001
Schools closed	Yes/No	31,530 (84.7%)	0	84.3%	85.9%	79.7%	$X_{\left(1\right)}^{2}$ = 204.83, *p* < 0.001
**Socio-demographics**							
Gender	Women	28,668 (77.1%)	0	73.6%			
	Men	8,539 (22.9%)		26.4%			
Age	Age given	36.70 (14.80)	0	36.72 (14.43)	36.20 (14.07)	38.18 (15.28)	*t*_15,846.15)_ = −11.44, *p* < 0.001
General financial struggles	1-7 Likert	2.90 (1.73)	0	2.91 (1.71)	2.90 (1.69)	2.96 (1.77)	*t*_16,235.03)_ = −3.11, *p* < 0.01
Financial struggles for food	1-7 Likert	2.50 (1.82)	0	2.48 (1.79)	2.44 (1.76)	2.59 (1.85)	*t*_16,200.54)_ = −6.98, *p* < 0.001
Loss of income	Yes / No	12,393 (33.3%)	4	33.6%	32.2%	37.6%	$X_{\left(1\right)}^{2}$ = 94.75, *p* < 0.001
Highest obtained degree			8				$X_{\left(1\right)}^{2}$ = 296.10, *p* < 0.001
Under a high school diploma		1,479 (4.0%)		4.3%	3.7%	6.2%	
High school diploma or equivalent		8,666 (23.3%)		24.9%	24.2%	26.6%	
Bachelor's degree		16,722 (45.0%)		40.6%	42.5%	35.1%	
Master's degree		8,040 (21.6%)		21.9%	22.1%	21.6%	
Doctorate		2,294 (6.2%)		8.3%	7.5%	10.5%	
Employment status during COVID-19			0				$X_{\left(1\right)}^{2}$ = 322.63, *p* < 0.001
Student		8,899 (23.9%)		23.4%	24.6%	20.2%	
Employed		18,096 (48.6%)		52.2%	49.4%	59.9%	
Not employed		10,212 (27.4%)		24.4%	26.0%	19.9%	
Number of cohabiting adults	Min 0 Max 12	2.38 (1.97)	343	2.26 (1.87)	2.30 (1.91)	2.16 (1.75)	*t*_18,322.07)_ = 6.33, *p* < 0.001
Number of cohabiting children	Min 0 Max 12	1.05 (1.44)	318	0.97 (1.41)	0.99 (1.41)	0.90 (1.41)	*t*_17,407.46)_ = 6.90, *p* < 0.001
Country of residence during COVID-19			0				
Australia		533 (1.4%)		2.6%	3.3%	0.8%	
Austria		362 (1%)		2.6%	3.0%	1.7%	
Bahrein		693 (1.9%)		2.6%	2.9%	1.8%	
Belgium		6,886 (18.5%)		2.6%	2.8%	2.0%	
Brazil		546 (1.5%)		2.6%	2.6%	2.7%	
Canada		844 (2.3%)		2.6%	2.9%	1.9%	
Chile		863 (2.3%)		2.6%	2.4%	3.1%	
China		539 (1.4%)		2.6%	1.4%	6.2%	
Denmark		835 (2.2%)		2.6%	1.7%	5.1%	
Ecuador		775 (2.1%)		2.6%	2.2%	3.7%	
Egypt		734 (2%)		2.6%	2.7%	2.3%	
Finland		791 (2.1%)		2.6%	3.3%	0.8%	
France		232 (0.6%)		2.6%	2.6%	2.8%	
Germany		662 (1.8%)		2.6%	2.1%	4.2%	
Greece		800 (2.2%)		2.6%	2.4%	3.4%	
Ireland		496 (1.3%)		2.6%	2.7%	2.4%	
Italy		315 (0.8%)		2.6%	2.9%	1.9%	
Japan		577 (1.6%)		2.6%	1.8%	4.8%	
Jordan		2,675 (7.2%)		2.6%	2.8%	2.2%	
Kuwait		728 (2.0%)		2.6%	2.8%	2.1%	
Lebanon		2,282 (6.1%)		2.6%	2.9%	1.9%	
Mexico		623 (1.7%)		2.6%	2.6%	2.6%	
Netherlands		778 (2.1%)		2.6%	2.9%	1.8%	
New Zealand		2,982 (8%)		2.6%	3.2%	1.0%	
Oman		186 (0.5%)		2.6%	3.0%	1.7%	
Palestine		859 (2.3%)		2.6%	2.8%	2.1%	
Peru		589 (1.6%)		2.6%	2.7%	2.4%	
Poland		550 (1.5%)		2.6%	2.0%	4.5%	
Qatar		653 (1.8%)		2.6%	2.8%	2.1%	
Romania		325 (0.9%)		2.6%	2.8%	2.1%	
Saudi Arabia		2,999 (8.1%)		2.6%	2.9%	1.8%	
Singapore		113 (0.3%)		2.6%	2.2%	3.7%	
South Africa		138 (0.4%)		2.6%	3.0%	1.5%	
Spain		730 (2%)		2.6%	2.7%	2.4%	
Uganda		320 (0.9%)		2.6%	1.8%	5.0%	
United Arab Emirates		1,718 (4.6%)		2.6%	2.9%	1.8%	
United Kingdom		205 (0.6%)		2.6%	2.5%	3.1%	
United States		271 (0.7%)		2.6%	2.7%	2.5%	

a Sample sizes of all participating countries differed. To control for over or underreporting from certain countries due to unequal survey collections, a survey weight created based on the country proportion in the total sample was applied in all analyses.

Valid percentage = responses only without considering missing values.

The Ethics Committee for the Social Sciences and Humanities of the University of Antwerp approved the study protocol (approval code 20_46).

### Participants

Eligible respondents were adults (18+ years old) residing in one of the 38 participating countries during the COVID-19 crisis. Respondents were recruited through convenience sampling; multiple banners were shared on social media, and the survey was advertised via several (inter)national press releases.

### Variables

The present study considered planning, selecting, and preparing healthier foods as part of the food literacy construct. Gender, age, educational attainment, employment status, income (for food), and the number of (adult) household members are considered as covariates of food literacy (4, 5, 7, 10, 17).

### Outcome Variables

Outcome variables were measured using 11 items from a validated food literacy scale that captures behaviors in the domains of “planning (and managing),” “selecting,” and “preparing” healthier foods (18). Answers were given on seven-point frequency scales (1 = never do this, to 7 = do this every time, see Table 1). Respondents were asked to answer each item twice, reporting their behavior before the COVID-19 crisis and at that moment (during the COVID-19 crisis). Variables (plan, select, prepare) were calculated following the original factor scores (18), and had high reliability scores in our sample (αFL_plan_before = 0.87, αFL_select_before = 0.84 αFL_prepare_before = 0.84; αFL_plan_during = 0.90, αFL_select_during = 0.89 αFL_prepare_during = 0.85). Change variables were computed by subtracting the before from the during scores. Changes in planning, selecting, and preparing healthier foods ranged from −6 to +6, with negative scores signifying a decline and positive scores indicating an increase.

### Predictors

Regarding predictors, psychological distress was measured with the Kessler K6 scale (19). The original scale assesses symptoms of psychological distress in the past 30 days. In this study, respondents indicated on seven-point frequency scales (1 = never to 7 = all the time) how often they experienced each of the six feelings since the COVID-19 crisis. The internal consistency of this scale in our sample was high (α = 0.88), and the mean sum score ranged from 1 (never distressed) to 7 (distressed all the time).

Financial stress was measured with a single item: “Since the COVID-19 crisis, I have experienced financial stress” answered on a seven-point frequency scale (1 = never to 7 = all the time). For time availability, respondents were asked how often since the COVID-19 crisis they felt they “had more time than usual,” using a similar seven-point frequency scale.

For social distancing policies, respondents answered yes (= 1) or no (= 0) to questions inquiring if they were forced to stay at/work from home, if public and private gatherings were forbidden or restricted, and if restaurants, pubs/bars, and schools were closed. We relied on self-report perceptions of social distancing measures rather than official announcements. This is because in many countries social distancing measures differed according to region and changed rapidly according to the situation. Moreover, in the end respondents' thoughts, feelings and behaviors may correspond more to what they believe is restricted rather than what is officially restricted or not.

### Control Variables

Modifying sociodemographic variables included gender, age, educational attainment, employment status, number of cohabiting adults and children, and general financial situation. Financial situation was measured with two questions: “In general, how often is it a struggle to make your money last until the end of the month/payday?” and “In general, how often is it a struggle to have enough money to go shopping for food?” using a seven-point frequency scale (1 = never to 7 = always). Loss of income was measured with the question “Have you lost (a part of your) income since the lockdown?” with answer options 1 = yes and 0 = no.

### Study Size and Statistical Analysis

Analyses were performed in SPSS version 26. Paired-samples *t*-test was first used to test the significance of changes in self-reported planning, selection, and preparation of healthier foods before vs. during COVID-19. Hierarchical multiple regression analyses were used to test the predicted model for planning, selecting, and preparing healthier foods as outcome measures separately. In all analyses feelings of psychological distress, financial stress, having more time than usual, and sociodemographic modifying variables were entered in the first block, and contextual factors in the second block, both by forced entry. Using G-power for this model with 17 predictors, anticipated small effect sizes of 0.01, and a level of significance set at a conservative *p* < 0.0001, a minimum total sample size of 4,881 was required. Missing values (see Table 1) were excluded listwise. A collinearity tolerance of < 0.20 and a VIF of five and above were used as criteria to control for multicollinearity. None of the reported regressions contained collinearity levels lower than 0.52 or VIF higher than 1.92, meaning no multicollinearity problems occurred.

Descriptive analyses, independent samples *t*-tests and chi-square tests (see Table 1) showed that scores of male and female respondents were different for all variables except for the perception of having more time. Gender differences in reported lockdown policies correspond to different gender ratios in the participating countries. Based on these results, all further analyses were performed separately for men and women. Sample sizes off all participating countries differed. To control for over or underreporting from certain countries due to unequal survey collections, a survey weight was created based on the country proportion in the total sample.

## Results

### Participants

Of all 81,486 people that started the survey, 38,666 completed the survey. Cases with invalid values for age (two cases) and gender (one case) were removed. Gender diverse (X-gendered) respondents (*n* = 128) were also omitted from analysis since this answer option was not used in every country, and the resulting subsample was too small for meaningful analyses. Respondents who did not live in one of the participating countries (*n* = 479) or did not provide their country of residence (*n* = 849) were also excluded from the analyses. A final *N* = 37,207 (73.6% women, *M*age = 36.72, *SD* = 14.43) were retained for analysis. Further details of the demographic characteristics of our sample are given in Table 1.

### Descriptive Results

Mean scores for planning, selecting, and preparing healthier foods were average to high before the COVID-19 crisis in both women and men. All three food literacy behavior domains increased during the COVID-19 crisis in both women and men [plan, women, *t*_27,381)_ = 40.11, *p* < 0.001, men *t*_9,824)_ = 16.909, *p* < 0.001; select, women, *t*_27,381)_ = 3.25, *p* < 0.01, men *t*_9,824)_ = 8.63, *p* < 0.001; prepare, women, *t*_27,381)_ = 27.58, *p* < 0.001, men *t*_9,824)_ = 9.47, *p* < 0.001, see Table 1 for all means and *SD*]. Furthermore, both men and women scored higher on financial stress when they had lost income due to COVID-19 [for women *t*_15,092.38)_ = 71.87, *p* < 0.001 with *M* = 2.35, *SD* = 1.48 for women who did not lose income and *M* = 3.89, *SD* = 1.74 for women who lost income; for men *t*_7,005.57)_ = 45.05, *p* < 0.001 with *M* = 2.38, *SD* = 1.53 for men who did not lose income and *M* = 3.95, *SD* = 1.74 for men who lost income].

### Hierarchical Multiple Regression Analyses

**Figure 1:**
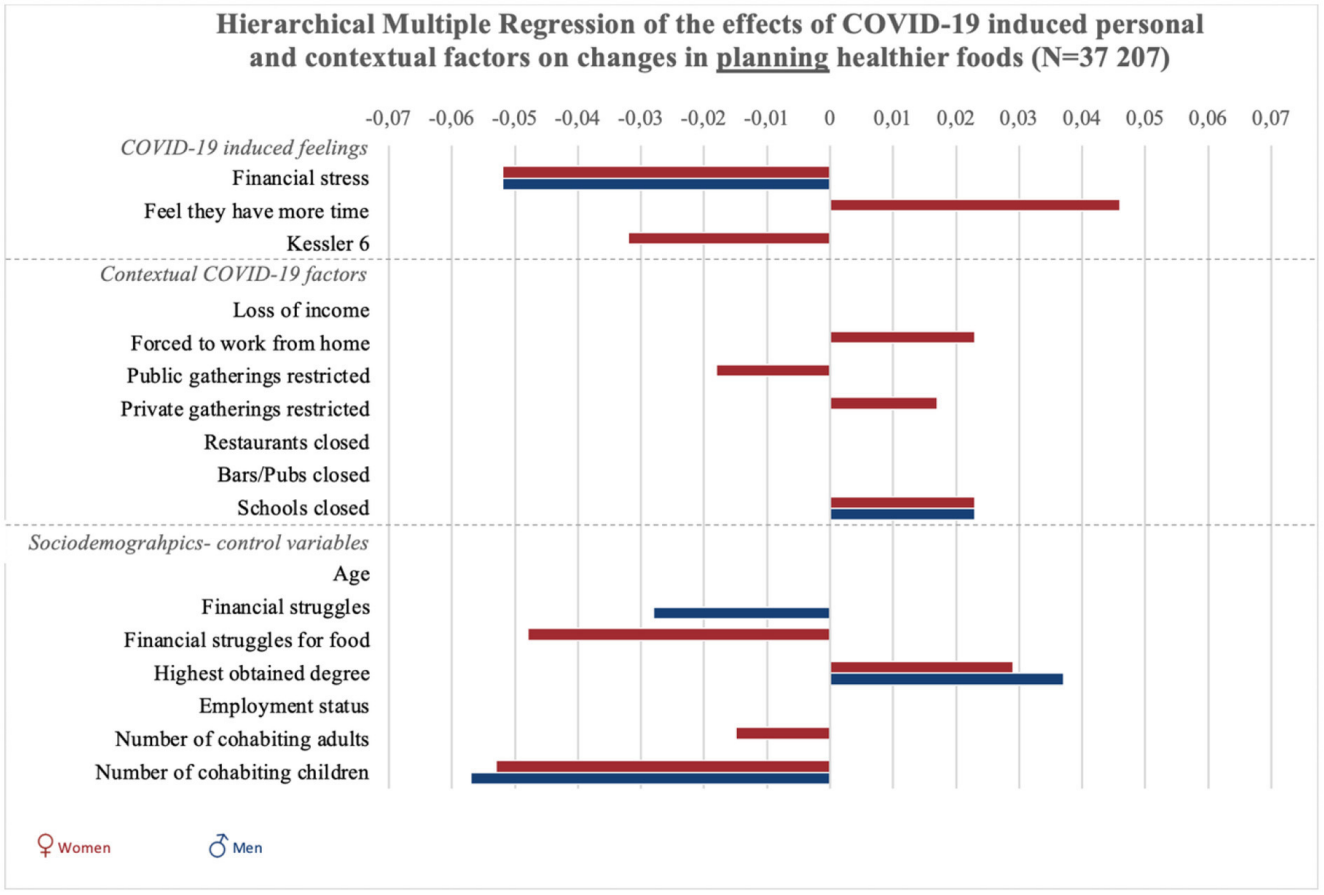
Graphic summary of the significant relations between personal, contextual and sociodemographic variables and changes in planning healthier foods during COVID-19. We report beta-values only for significant relations in models 2 for planning healthier foods. Bars to the right indicate improvement in food planning, bars to the left indicate decreases in planning healthy foods.

**Figure 2:**
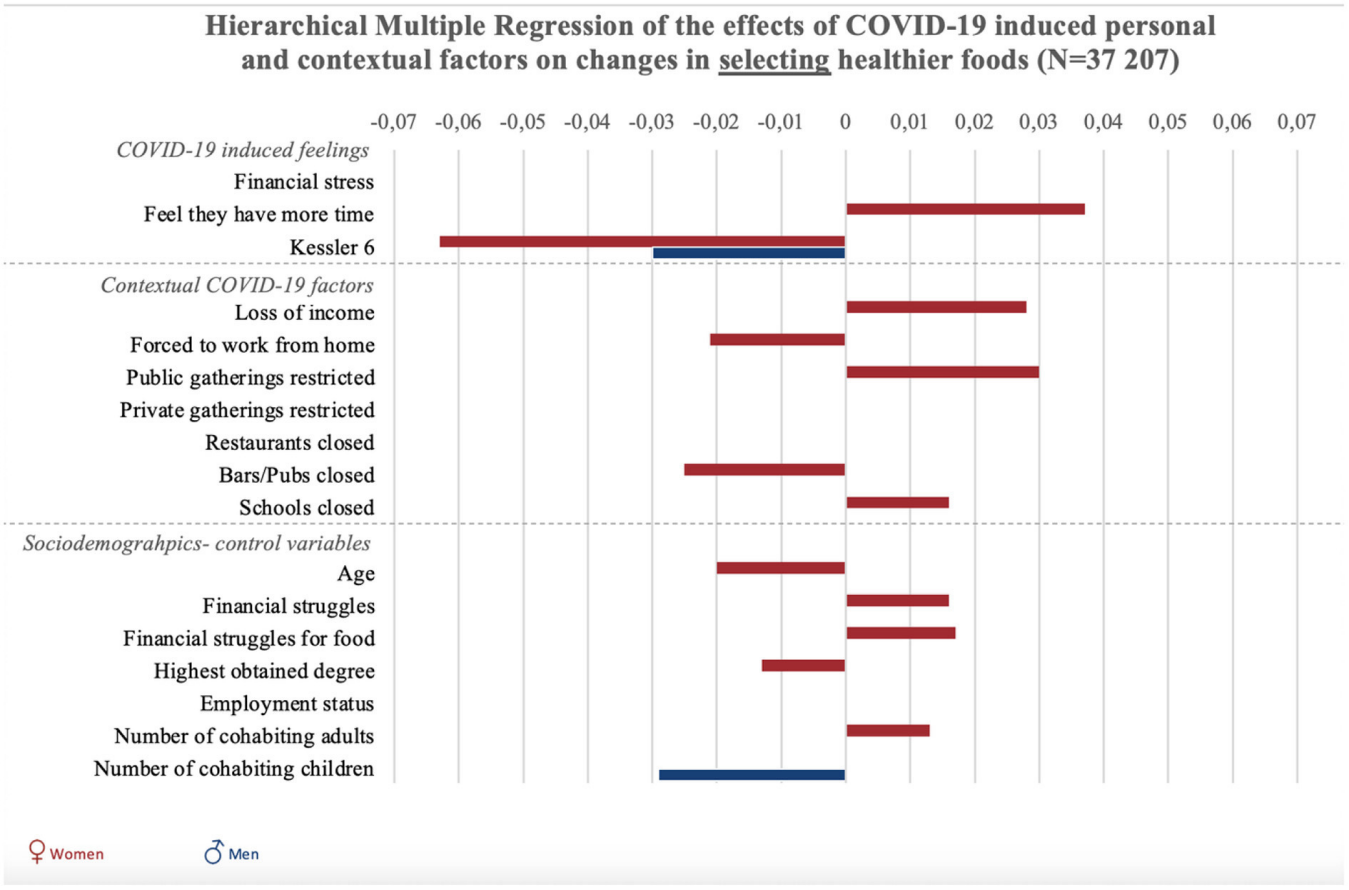
Graphic summary of the significant relations between personal, contextual and sociodemographic variables and changes in selecting healthier foods during COVID-19. We report beta-values only for significant relations in models 2 for selecting healthier foods. Bars to the right indicate improvement in food selection, bars to the left indicate decreases selecting healthy foods.

**Figure 3:**
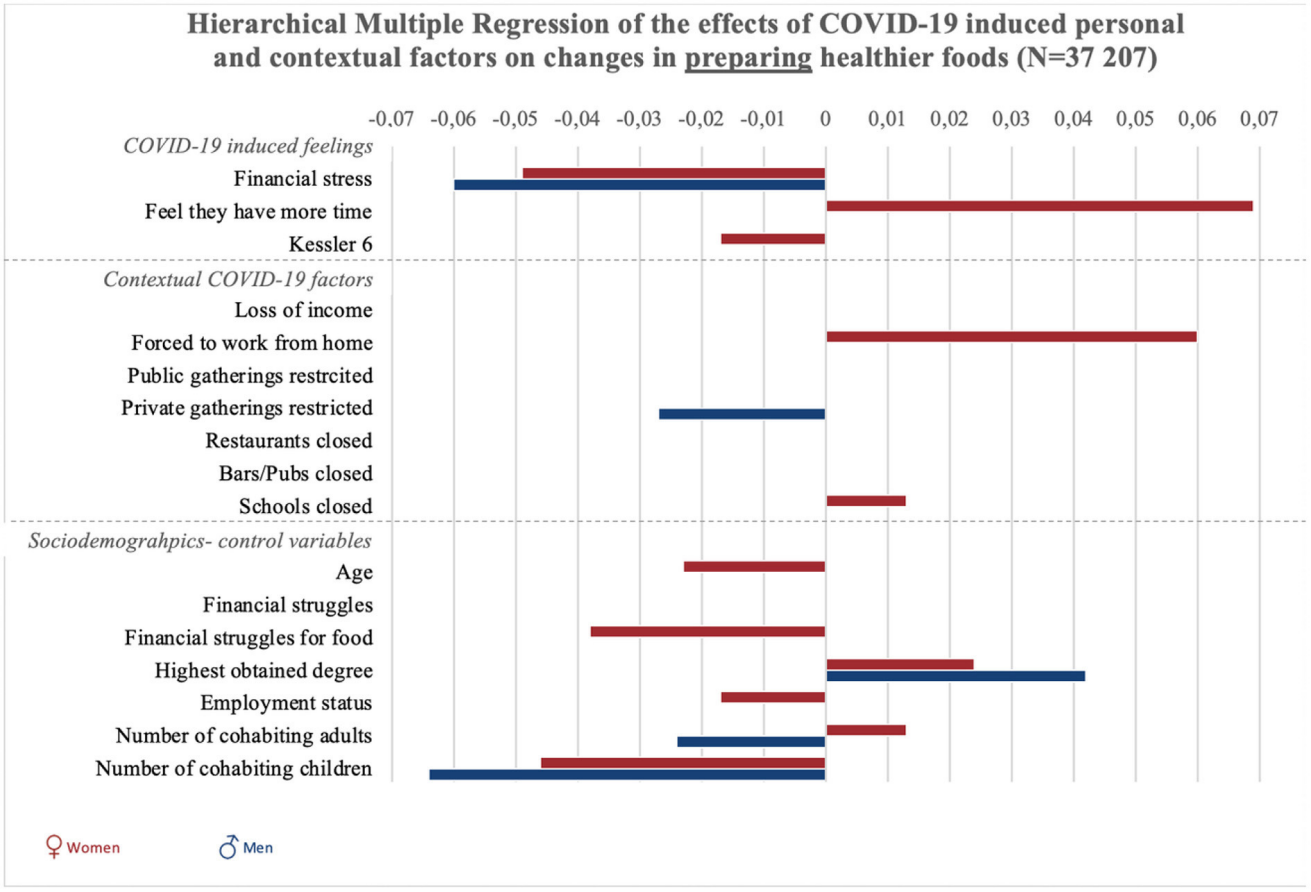
Graphic summary of the significant relations between personal, contextual and sociodemographic variables and changes in preparing healthier foods during COVID-19. We report beta-values only for significant relations in models 2 for preparing healthier foods. Bars to the right indicate improvement in food preparation, bars to the left indicate decreases in preparing healthy foods.

Results of all hierarchical multiple regression analyses are reported in full detail in Supplementary Table 2, and summarized in Figures 1, 2 and 3. To start with the personal responses, the perception of having more time since the COVID-19 crisis was associated with increases in planning, selecting, and preparing healthier foods in women (*p* < 0.001), but not significantly in men (*p* = 0.54). COVID-19-induced financial stress was associated with decreases in planning and preparing healthier foods in both women and men (*p* < 0.001). COVID-19-induced psychological distress was associated with decreases in planning, selecting, and preparing healthier foods among women (*p* < 0.05). For men, psychological distress was negatively related to selecting healthier foods (*p* < 0.05).

Concerning contextual factors, positive associations were found between policies to stay at home/work from home and changes in planning and preparing healthier foods in women (*p* < 0.001). However, staying home was negatively associated with selecting healthier foods in women (*p* < 0.01). Next, policies on public gatherings related to an increase in selecting healthier foods among women (*p* < 0.01). Policies on public gatherings also negatively related to women's planning of healthier foods (*p* < 0.05). Policies on private gatherings positively related to women's planning (*p* < 0.05), and was negatively related to men's preparation of healthier foods (*p* < 0.05).

The closure of schools was associated with increased healthier food planning in men and women, as well as selection and preparation in women (*p* < 0.05). The closure of pubs and bars was associated with decreases in selecting healthier foods in women (*p* < 0.001).

Regarding the sociodemographic characteristics associated with changes in food literacy behaviors, educational attainment was negatively related to changes in selecting healthier foods in women (*p* < 0.05) and positively related to changes in planning and preparing healthier foods in men and women (*p* < 0.001). Employment status was negatively related to changes in food preparation in women (*p* < 0.05). Struggling to make money last until the next payday was positively related to changes in women's selecting healthier foods (*p* < 0.05), and negatively related to men's changes in food planning (*p* < 0.05). Struggling to have enough money to go shopping for food was also related to positive changes in women's use of food labels (selecting healthier foods), but related to negative changes in women's planning and preparing healthier foods (*p* < 0.01). Also loss of income was related to an increase in selecting healthier foods among women (*p* < 0.001). For women, age was negatively related to changes in selecting and preparing healthier foods (*p* < 0.01). Finally, the more adult cohabitants men had during the COVID-19 crisis, the more their preparation of healthier foods decreased (*p* < 0.01). For women, increases in the number of adult cohabitants related to decreases in planning healthier foods (*p* < 0.05). The number of children in the household was negatively associated with men and women's planning and preparation of healthier foods (*p* < 0.001), and also negatively associated with men's selection of healthier foods (*p* < 0.01).

## Discussion

Observations from this study in 38 countries worldwide during the COVID-19 crisis show that positive changes in food literacy can be achieved, and often depend on combinations of personal characteristics and circumstances. Three key learnings from the available evidence are useful in informing future nutrition interventions.

First, the COVID-19 crisis has taught us that stay-at-home policies, and especially personal perceptions of having more time among women, can increase the willingness to plan, select, and prepare healthier foods. Stay-at-home policies resulted in distorted perceptions of time and made many people feel bored (12, 13). Yet, stay-at-home policies may be in our favor when it comes to food literacy, if people feel to have more time, because in these cases we observed positive increases in planning, preparing, and selecting healthier foods among women. A health equity lens is warranted (3), however, since working from home is not beneficial for everyone and can lead to increased stress in some people (20). This is reflected in our results showing that while feeling to have more time relates to increases in planning, selecting, and preparing healthier foods among women, stay-at-home policies corresponded to decreases in selecting healthier foods as well among this group. These seemingly contradicting results can perhaps be brought back to time perception, as time constraints are an important factor in practicing healthy food behaviors (21). Stay-at-home policies specifically could be responsible for this dual outcome of either experiencing more or less time constraints, as some have experienced having more time during COVID-19 work from home obligations (13), and others—mainly parents and mothers especially—have had less or more fragmented time perceptions (22). Mothers during COVID-19 have especially perceived more time-related stress in combing their work and home responsibilities (22), aligning with previous findings that women with young children in particular experience more stress and time constraints when working from home (23). We also observed that an increase in the number of children one lives with relates to a decrease in changes in planning and preparing healthier foods in men and women, as well as selecting them for men. Thus, health practitioners should find ways of incorporating workplace policies to increase time availability in long-term food literacy interventions, bearing the home situation in mind for parents and especially mothers. The requirement to work from home has been a successful public health initiative to curb the spread of COVID-19, and may be a successful long-term strategy to improve food literacy, other factors considered.

Second, nutrition interventions should also be cognizant of mental health and focus on strategies to deal with psychological distress, especially among women. The COVID-19 crisis caused considerable distress (11); our results show that women experienced more psychological distress compared to men. Furthermore, women's psychological distress was related to decreased planning, selection, and preparation of healthier foods. Increases in psychological distress have been linked to averse nutritional health behaviors in the past (24). Previous studies have highlighted different possible causes to increased distress as a result of COVID-19 lockdown. Some studies have cited the distorted time perceptions and a sense of timelessness as a possible cause for sadness psychological distress (12, 13). Others cite lower socioeconomic status, COVID-19 infection risk, and longer media exposure as factors related to psychological distress (25). Women especially have been associated with higher psychological distress (25), which could explain our findings as they related to food literacy behaviors.

Third, our results confirm that policymakers must apply a health equity lens, and see both overt and subtle social differences (24). For instance, they should not only focus on income, but on personal feelings of financial stress as well. Our results show that COVID-19 induced loss of income relates to significantly higher levels of financial stress. Loss of income and struggling to have enough money for food related to increases in selecting healthier foods for women. When looking at the planning and preparation of healthier meals, however, results show a different pattern: financial stress related to decreases in planning and preparing healthier meals for both men and women, whereas struggles to have enough money for food related to these decreases only among women. Thus, while financial stress and -constraints decreased women's planning and preparation of healthier meals, it seemed to increase their selection of healthy meals. A potential explanation for this may be found in grocery shopping as it relates to meal selection, as prices of certain foods became more expensive, especially for foods that were hoarded due to social panic (26). If one needs to switch to more expensive alternatives, increased attention to food labels may occur. Consumers subscribe to a general lay theory that more expensive foods equal to healthier foods (27). This could also explain our other finding, that during the COVID-19 crisis, food labels were mostly read by lower-educated women. However, this may have occurred not necessarily because of an increased knowledge-driven interest, but because of a more critical attitude when having to (relatively) spend more money on food. Previous studies on effects of crisis periods on cooking patterns also demonstrated mixed results (28–30). Our results confirm that the relation between economic constraints and unhealthy food habits is complex (31): a lack of money (for food) and the accompanying stress diversely relate to how people select, plan, and prepare food.

With regard to other sociodemographic characteristics, our results show that increases in food planning were associated with older age in men and women, while, for women, age was related negatively to changes in selecting and preparing healthier foods. A potential explanation for this is that more women acquire higher levels of food literacy at a younger age than men, leaving less room for improvement as they get older (4, 5, 7, 10). Additionally, these results can be linked to younger age being associated with increased psychological distress during COVID-19 (25), potentially causing less healthy food behaviors (24).

This study has several strengths. First, we reported changes in planning, selecting, and preparing healthier foods during the COVID-19 crisis in 38 countries worldwide. International collaborations are important to understand food literacy within the complex context of ecological influences (32). Our results confirm that the COVID-19 crisis is related to changes in food and nutrition, as was expected (11). Second, by inquiring about food literacy behavior both before and during the COVID-19 crisis with short validated instruments (18), we could control for baseline (pre-COVID-19) levels of food literacy behavior, which were generally average too high in our sample. Third, we gathered information on known personal factors and a range of suspected contextual factors that fluctuated. Social distancing policies were enforced in some, but not all, of the participating countries, and even within one country regional differences applied. This yielded sufficient variation to test for the effects of specific lockdown policies. Finally, there is limited empirical research concerning both intrinsic and extrinsic factors related to food literacy in general (4, 5, 7, 8, 10). This is the first empirical study that looked at factors that can facilitate or impede aspects of food literacy in 38 countries worldwide.

We acknowledge several limitations. First, we looked at planning, selecting, and preparing healthier foods as components of food literacy. Food literacy consists of personal skills, knowledge, self-efficacy, beliefs, feelings, and behavior, which interplay with contextual factors (4, 5, 7, 10). These various complex factors make food literacy a difficult concept to measure, and a scale that captures all food literacy aspects does not currently exist. A second limitation was the small effect sizes. Small effect sizes are more likely in large (*N* ≥ 2,000) and heterogeneous samples, where there is a lot of variation in context that affects how easily the dependent variable can be influenced (33). Changing food literacy is difficult (4, 5, 7, 10), and our sample of *N* = 37,203 was very heterogeneous, covering 38 countries worldwide. Finally, our sample was not achieved through a random sampling of populations; there was a clear overrepresentation of women and highly educated people. Our sample size was large enough to achieve valid results for all groups, but in future planned data collections, we will need greater targeted outreach of underrepresented populations.

In conclusion, we reported overall increases in planning, selecting, and preparing healthier foods during the COVID-19 crisis among women and men in 38 countries around the world using self-report data. Perceptions of having more time were most clearly associated with these positive changes among women, followed by the contextual factor of stay-at-home policies. Psychological distress was related to decreases in women's food literacy, and decreases in men's healthy food selection. Financial stress was not always related to decreases in food literacy, financial stress and struggles related to increased healthier food selection behaviors among women but decreased in planning and preparing.

## Data Availability Statement

The raw data supporting the conclusions of this article will be made available by the authors, without undue reservation.

## Ethics Statement

The studies involving human participants were reviewed and approved by Ethical Committee for the Social Sciences and Humanities of the University of Antwerp. File number 20_46. The patients/participants provided their written informed consent to participate in this study.

## Author Contributions

The study was conceptualized by CD, LT, IC, PD, SP, and KV. Data were collected by all members of the Corona Cooking Survey Study Group. Data analysis was done by CD. The original manuscript was prepared by CD, LT, IC, PD, SP, CM, HA, and KV. Further writing, reviewing and editing was done in multiple rounds by the entire Corona Cooking Survey Study Group. All authors have read and agreed to the final version of the manuscript.

## Conflict of Interest

The authors declare that the research was conducted in the absence of any commercial or financial relationships that could be construed as a potential conflict of interest.

## Publisher's Note

All claims expressed in this article are solely those of the authors and do not necessarily represent those of their affiliated organizations, or those of the publisher, the editors and the reviewers. Any product that may be evaluated in this article, or claim that may be made by its manufacturer, is not guaranteed or endorsed by the publisher.

## Footnotes

^1^Australia; Austria; Bahrein; Belgium; Brazil; Canada; Chile; China; Denmark; Ecuador; Egypt; Finland; France; Germany; Greece; Ireland; Italy; Japan; Jordan; Kuwait; Lebanon; Mexico; Netherlands; New Zealand; Oman; Palestine; Peru; Poland; Qatar; Romania; Saudi Arabia; Singapore; South Africa; Spain; Uganda; United Arab Emirates; United Kingdom; United States.

## Corona Cooking Survey Study Group

The Corona Cooking Survey Study Group consists of (per country) **Austria:** Ina Bergheim, Raphaela Staltner (University of Vienna); **Australia:** Amanda Devine, Ros Sambell, Ruth Wallace (Edith Cowan University); **Bahrein:** Sabika Salem Allehdan (University of Jordan), Tariq Abdulkarim Alalwan, Mariam Ahamad Al-Mannai (University of Bahrain), Leila Cheikh Ismail (Sharjah University); **Belgium:** Charlotte De Backer, Kathleen Van Royen, Lauranna Teunissen, Isabelle Cuykx, Paulien Decorte, Gaelle Ouvrein, Karolien Poels, Heidi Vandebosch, Katrien Maldoy (University of Antwerp); Sara Pabian (Tilburg University), Christophe Matthys, Tim Smits, Jules Vrinten (KULeuven); Ann DeSmet (University of Antwerp, Universite Libre de Bruxelles); Nelleke Teughels, Maggie Geuens, Iris Vermeir, Viktor Proesmans, Liselot Hudders (Ghent University); **Brazil:** Marcia Dutra De Barcellos, Cristina Ostermann, Ana Luiza Brock, Cynthia Favieiro, Rafaela Trizotto, Isadora Stangherlin (Universidade Federal do Rio Grande do Sul); Anthonieta Looman Mafra, Marco Antonio Correa Varella, Jaroslava Varella Valentova (University of São Paulo); **Canada:** Maryanne L. Fisher (Saint Mary's University), Melanie MacEacheron (Arizona State University), Katherine White, Rishad Habib (The University of British Columbia), David S. Dobson (University of the Fraser Valley); **Chile:** Berta Schnettler, Ligia Orellana, Edgardo Miranda-Zapata (Universidad de La Frontera); **China:** Angela Wen-Yu Chang, Wen Jiao, Matthew Tingchi, Liu (University of Macau); **Denmark:** Klaus G. Grunert, Rikke Nyland Christensen (Aarhus University) Lucia Reisch, Meike Janssen (Copenhagen Business School); **Ecuador:** Victoria Abril-Ulloa, Lorena Encalada (Universidad de Cuenca); **Egypt:** Iman Kamel (National Research Centre Egypt); **Finland:** Annukka Vainio, Mari Niva, Laura Salmivaara, Johanna Makela, Kaisa Torkkeli (University of Helsinki); **France:** Robert Mai (Grenoble Ecole de Management); **Germany:** Pamela Kerschke-Risch (University of Hamburg); **Greece:** Efthymios Altsitsiadis (Copenhagen Business School), Angelos Stamos (University of Northumbria), Andreas Antronikidis (University of Macedonia), Stelios Tsafarakis, Pavlos Delias **Iran:** Hamid Rasekhi, Mohammad Reza Vafa, Karandish Majid, Hassan Eftekhari (Ahvaz Jundishapur University of Medical Sciences); **Ireland:** Maeve Henchion, Sinead McCarthy (Teagasc), Mary McCarthy (University College Cork); **Italy:** Alessandra Micalizzi (SAE Institute of Milan), Peter J. Schulz (University of Lugano), Manuela Farinosi (University of Udine); **Japan:** Hidenori Komatsu, Nobuyuki Tanaka, Hiromi Kubota (Central Research Institute of Electric Power Industry); **Jordan:** Reema Tayyem (The University of Jordan), Narmeen J. Al-Awwad (The Hashemite University), Nahla Al-Bayyari (Al-Balqa Applied University), Mohammed O. Ibrahim (Muftah University), Fadwa Hammouh (American University of Madaba); **Kuwait:** Somaia Dashti (Public Authority for Applied Education & Training), Basma Dashti (Kuwait Institute for Scientific Research), Dhuha Alkharaif (Public Authority for Applied Education & Training), Amani Alshatti (Public Authority for Applied Education & Training), Maryam Al Mazedi (Public Authority for Applied Education & Training); **Lebanon:** Maha Hoteit, Rania Mansour, Elissa Naim, Hussein Mortada (Lebanese University); **Mexico:** Yareni Yunuen Gutierrez Gomez (Tecnologico de Monterrey); **Netherlands:** Kelly Geyskens, Caroline Goukens (Maastricht University); **New Zealand:** Sarah Gerritsen, Rajshri Roy, Victoria Egli (University of Auckland), Lisa Te Morenga (Victoria University of Wellington); **Oman:** Mostafa Waly (Sultan Qaboos University); **Palestine:** Radwan Qasrawi, Motasem Hamdan, Rania Abu Sier, Diala Abu Al Halawa, Haleama Al Sabbah, Hazem Agha (Al Quds University); **Peru:** Maria Reyna Liria-Dominguez (Instituto de Investigacion Nutricional/Universidad Peruana de Ciencias Aplicadas), Lita Palomares (Universidad Peruana Cayetano Heredia); **Poland:** Gra.yna W.sowicz (Kozminski University); **Qatar:** Hiba Bawadi, Manal Othman (Hamad Medical Corporation), Jaafar Pakari, Allam Abu Farha, Rasha Abu-El-Ruz (Qatar University; QU Health); **Romania:** Dacinia Crina Petrescu, Ruxandra Malina Petrescu-Mag (Babes-Bolyai University); Felix Arion (University of Agricultural Sciences and Veterinary Medicine of Cluj-Napoca), Stefan Cristian Vesa (Iuliu Hatieganu University of Medicine and Pharmacy); **Saudi Arabia:** Majid M. Alkhalaf (National Nutrition Committee (NNC) at Saudi Food and Drug Authority (Saudi FDA)), Khlood Bookari (Taibah University), Jamila Arrish (National Nutrition Committee (NNC) at Saudi Food and Drug Authority (Saudi FDA)); **Singapore:** Zackaria Rahim, Roy Kheng (Institution); **South Africa:** Yandisa Ngqangashe, Zandile June-Rose Mchiza (University of the Western Cape); **Spain:** Marcela Gonzalez-Gross, Lisset Pantoja-Arevalo, Eva Gesteiro (Universidad Politecnica de Madrid); Yolanda Rios (AZTI, Food Research, Basque Research and Technology Alliance (BRTA)); **Uganda:** Peter Yiga, Patrick Ogwok, Denis Ocen (Kyambogo University), Michael Bamuwamye (Kyambogo University/Ministry of Health Uganda); **United Arab Emirates:** Haleama Al Sabbah, Zainab Taha (Zayed University), Leila Cheikh Ismail (Sharjah University), Ayesha Aldhaheri (United Arab Emirates University); **United Kingdom:** Elisa Pineda, Marisa Miraldo (Imperial College London); Dawn Liu Holford (University of Essex); **United States:** Hilde Van den Bulck (Drexel University).
