# Corrigendum: An evaluation of the COVID-19 pandemic and perceived social distancing policies in relation to planning, selecting, and preparing healthy meals: An observational study in 38 countries worldwide

**DOI:** 10.3389/fnut.2022.989617

**Published:** 2022-10-17

**Authors:** Charlotte De Backer, Lauranna Teunissen, Isabelle Cuykx, Paulien Decorte, Sara Pabian, Sarah Gerritsen, Christophe Matthys, Haleama Al Sabbah, Kathleen Van Royen, the Corona Cooking Survey Study Group

**Affiliations:** ^1^Department of Communication Sciences, Faculty of Social Sciences, University of Antwerp, Antwerp, Belgium; ^2^Tilburg Center for Cognition and Communication, Tilburg School of Humanities and Digital Sciences, Tilburg University, Tilburg, Netherlands; ^3^School of Population Health, University of Auckland, Auckland, New Zealand; ^4^Clinical and Experimental Endocrinology, KU Leuven, Leuven, Belgium; ^5^Public Health Nutrition Department, Zayed University, Dubai, United Arab Emirates; ^6^Family Medicine and Population Health, Faculty of Medicine and Health Sciences, University of Antwerp, Antwerp, Belgium

**Keywords:** food literacy, food planning, food preparation, food selection, nutrition, COVID-19, psychological distress, time availability

In the original article, there was an error in weighting the data for the statistical analyses. The data was re-analyzed carefully using the correct weighting coefficients based on the country proportion in the total sample, to correctly control for underreporting from certain countries due to unequal survey collections.

The key message of the published article remains the same, namely that food literacy in terms of selecting, preparing, and planning of healthy foods increased during COVID-19 lockdown among both women and men. Additionally, the perceived time availability and stay-at-home policies remain positively associated with increased food literacy levels, however only for women and not for men. Also, staying at home policies remains negatively associated with selecting healthier foods for women, though it is no longer significant for men.

The new analyses with the correct weighting coefficients impact the **Abstract**, **Materials and Methods** (“*Study Size and Statistical Analysis”*), **Results** and **Discussion** sections, therefore corrections have been made. However, the list of adjustments appears more extensive than it actually is. All corrections are summed up per section.

**Corrections to text, figures and tables due to incorrect use of weighting coefficient**

Corrections have been made to the **Abstract**.

The original Methods section of the Abstract stated:

“Using cross-sectional online surveys collected in 38 countries worldwide in April-June 2020 (*N* = 37,207, *M*age 36.7 *SD* 14.8, 77% women), we compared changes in food literacy behaviors to changes in personal factors and social distancing policies, using hierarchical multiple regression analyses controlling for sociodemographic variables.”

The correct paragraph is stated below:

“Using cross-sectional online surveys collected in 38 countries worldwide in April-June 2020 (*N* = 37,207, *M*age 36.7 *SD* 14.43, 73.6% women), we compared changes in food literacy behaviors to changes in personal factors and social distancing policies, using hierarchical multiple regression analyses controlling for sociodemographic variables.”

The original Results section of the Abstract stated:

“Increases in planning (4.7 *SD* 1.3, 4.9 *SD* 1.3), selecting (3.6 *SD* 1.7, 3.7 *SD* 1.7), and preparing (4.6 *SD* 1.2, 4.7 *SD* 1.3) healthy foods were found for women and men, and positively related to perceived time availability and stay-at-home policies. Psychological distress was a barrier for women, and an enabler for men. Financial stress was a barrier and enabler depending on various sociodemographic variables (all *p* < 0.01).”

The correct paragraph is stated below:

“Increases in planning (4.7 *SD* 1.2, 4.9 *SD* 1.3), selecting (3.8 *SD* 1.7, 3.8 *SD* 1.7), and preparing (4.6 *SD* 1.3, 4.7 *SD* 1.3) healthy foods were found for women and men, and positively related to perceived time availability among women and stay-at-home policies for planning and preparing in women. Psychological distress was a barrier for women, and an enabler for men. COVID-19 induced financial stress was a barrier depending on various sociodemographic variables (all *p* < 0.01).”

The original Conclusion section of the Abstract stated:

“Stay-at-home policies and feelings of having more time during COVID-19 seem to have improved food literacy. Stress and other social distancing policies relate to food literacy in more complex ways, highlighting the necessity of a health equity lens.”

The correct paragraph is stated below:

“Stay-at-home policies and feelings of having more time during COVID-19 seem to have improved food literacy among women. Stress and other social distancing policies relate to food literacy in more complex ways, highlighting the necessity of a health equity lens.”

Corrections have been made to the section **Materials and Methods**, “*Study Size and Statistical Analysis*,” paragraph 2. The first correction was made to the sentence that previously stated:

“Descriptive analyses, independent samples *t*-tests and chi-square tests (see Table 1) showed that scores of male and female respondents were different for all variables except for the perception of having more time and general financial struggles.”

**Table 1:** Detailed descriptive statistics (Means, Standard Deviations, and Valid Percentages) for the entire sample, weighted^a^ and subsamples of women and men, used in all analyses.

		**Total sample *N* = 37,207**		**Weighted sample used in analyses**	**Weighted female subsample**	**Weighted male subsample**	
	**Answer option**	***M*** **(*****SD*****) or** ***n*** **(valid %)**	**Missing values** ***n***	***M*** **(*****SD*****)** **or valid %**	***M*** **(*****SD*****)** **or valid %**	***M*** **(*****SD*****)** **or valid %**	**Significance of sex**. **differences based on** ***t*****-tests (*****M***, ***SD*****) or Chi-square (%)**
**Food literacy scores**							
Plan before COVID-19	1–7 Likert	4.70 (1.26)	0	4.66 (1.24)	4.77 (1.21)	4.36 (1.28)	*t*_16,156.28)_ = 28.33, *p* < 0.001
Plan during COVID-19	1–7 Likert	4.89 (1.34)	0	4.87 (1.31)	5.00 (1.27)	4.51 (1.36)	*t*_15,928.77)_ = 31.47, *p* < 0.001
Select before COVID-19	1–7 Likert	3.61 (1.65)	0	3.75 (1.66)	3.84 (1.66)	3.53 (1.66)	*t*_36,654)_ = 16.40, *p* < 0.001
Select during COVID-19	1–7 Likert	3.66 (1.71)	0	3.80 (1.71)	3.86 (1.71)	3.62 (1.68)	*t*_36,654)_ = 12.32, *p* < 0.001
Prepare food before COVID-19	1–7 Likert	4.60 (1.24)	0	4.56 (1.25)	4.69 (1.20)	4.22 (1.33)	*t*_15,486.65)_ = 30.64, *p* < 0.001
Prepare food during COVID-19	1–7 Likert	4.72 (1.29)	0	4.71 (1.29)	4.85 (1.23)	4.31 (1.38)	*t*_15,471.12)_ = 34.08, *p* < 0.001
**COVID-19 induced feelings**							
Financial stress	1–7 Likert	2.85 (1.76)	0	2.88 (1.74)	2.85 (1.73)	2.97 (1.78)	*t*_16,581.94)_ = −5.234, *p* < 0.001
Feel they have more time	1–7 Likert	4.18 (1.74)	0	4.15 (1.75)	4.15 (1.75)	4.17 (1.74)	*t*_36,654)_ = −1.183, *p* = 0.237
KESSLER 6	1–7 Likert	3.06 (1.28)	0	3.07 (1.26)	3.15 (1.25)	2.86 (1.26)	*t*_36,654)_ = 20.52, *p* < 0.001
**COVID-19 contextual factors**
Forced to work/stay home	Yes/No	29,558 (79.4%)	0	80.5%	82.00%	76.2%	$X_{\left(1\right)}^{2}$ = 154.74, *p* < 0.001
Public gatherings restricted	Yes/No	9,464 (25.4%)	0	27.1%	25.9%	30.5%	$X_{\left(1\right)}^{2}$ = 78.90, *p* < 0.001
Private gatherings restricted	Yes/No	5,508 (14.8%)	0	14.9%	14.3%	16.4%	$X_{\left(1\right)}^{2}$ = 25.75, *p* < 0.001
Restaurants closed	Yes/No	28,309 (76.1%)	0	77.4%%	79.1%	72.7%	$X_{\left(1\right)}^{2}$ = 168.79, *p* < 0.001
Bars/pubs closed	Yes/No	29,259 (78.6%)	0	79.7%	80.2%	78.3%	$X_{\left(1\right)}^{2}$ = 16.08, *p* < 0.001
Schools closed	Yes/No	31,530 (84.7%)	0	84.3%	85.9%	79.7%	$X_{\left(1\right)}^{2}$ = 204.83, *p* < 0.001
**Socio-demographics**							
Gender	Women	28,668 (77.1%)	0	73.6%			
	Men	8,539 (22.9%)		26.4%			
Age	Age given	36.70 (14.80)	0	36.72 (14.43)	36.20 (14.07)	38.18 (15.28)	*t*_15,846.15)_ = −11.44, *p* < 0.001
General financial struggles	1-7 Likert	2.90 (1.73)	0	2.91 (1.71)	2.90 (1.69)	2.96 (1.77)	*t*_16,235.03)_ = −3.11, *p* < 0.01
Financial struggles for food	1-7 Likert	2.50 (1.82)	0	2.48 (1.79)	2.44 (1.76)	2.59 (1.85)	*t*_16,200.54)_ = −6.98, *p* < 0.001
Loss of income	Yes / No	12,393 (33.3%)	4	33.6%	32.2%	37.6%	$X_{\left(1\right)}^{2}$ = 94.75, *p* < 0.001
Highest obtained degree			8				$X_{\left(1\right)}^{2}$ = 296.10, *p* < 0.001
Under a high school diploma		1,479 (4.0%)		4.3%	3.7%	6.2%	
High school diploma or equivalent		8,666 (23.3%)		24.9%	24.2%	26.6%	
Bachelor's degree		16,722 (45.0%)		40.6%	42.5%	35.1%	
Master's degree		8,040 (21.6%)		21.9%	22.1%	21.6%	
Doctorate		2,294 (6.2%)		8.3%	7.5%	10.5%	
Employment status during COVID-19			0				$X_{\left(1\right)}^{2}$ = 322.63, *p* < 0.001
Student		8,899 (23.9%)		23.4%	24.6%	20.2%	
Employed		18,096 (48.6%)		52.2%	49.4%	59.9%	
Not employed		10,212 (27.4%)		24.4%	26.0%	19.9%	
Number of cohabiting adults	Min 0 Max 12	2.38 (1.97)	343	2.26 (1.87)	2.30 (1.91)	2.16 (1.75)	*t*_18,322.07)_ = 6.33, *p* < 0.001
Number of cohabiting children	Min 0 Max 12	1.05 (1.44)	318	0.97 (1.41)	0.99 (1.41)	0.90 (1.41)	*t*_17,407.46)_ = 6.90, *p* < 0.001
Country of residence during COVID-19			0				
Australia		533 (1.4%)		2.6%	3.3%	0.8%	
Austria		362 (1%)		2.6%	3.0%	1.7%	
Bahrein		693 (1.9%)		2.6%	2.9%	1.8%	
Belgium		6,886 (18.5%)		2.6%	2.8%	2.0%	
Brazil		546 (1.5%)		2.6%	2.6%	2.7%	
Canada		844 (2.3%)		2.6%	2.9%	1.9%	
Chile		863 (2.3%)		2.6%	2.4%	3.1%	
China		539 (1.4%)		2.6%	1.4%	6.2%	
Denmark		835 (2.2%)		2.6%	1.7%	5.1%	
Ecuador		775 (2.1%)		2.6%	2.2%	3.7%	
Egypt		734 (2%)		2.6%	2.7%	2.3%	
Finland		791 (2.1%)		2.6%	3.3%	0.8%	
France		232 (0.6%)		2.6%	2.6%	2.8%	
Germany		662 (1.8%)		2.6%	2.1%	4.2%	
Greece		800 (2.2%)		2.6%	2.4%	3.4%	
Ireland		496 (1.3%)		2.6%	2.7%	2.4%	
Italy		315 (0.8%)		2.6%	2.9%	1.9%	
Japan		577 (1.6%)		2.6%	1.8%	4.8%	
Jordan		2,675 (7.2%)		2.6%	2.8%	2.2%	
Kuwait		728 (2.0%)		2.6%	2.8%	2.1%	
Lebanon		2,282 (6.1%)		2.6%	2.9%	1.9%	
Mexico		623 (1.7%)		2.6%	2.6%	2.6%	
Netherlands		778 (2.1%)		2.6%	2.9%	1.8%	
New Zealand		2,982 (8%)		2.6%	3.2%	1.0%	
Oman		186 (0.5%)		2.6%	3.0%	1.7%	
Palestine		859 (2.3%)		2.6%	2.8%	2.1%	
Peru		589 (1.6%)		2.6%	2.7%	2.4%	
Poland		550 (1.5%)		2.6%	2.0%	4.5%	
Qatar		653 (1.8%)		2.6%	2.8%	2.1%	
Romania		325 (0.9%)		2.6%	2.8%	2.1%	
Saudi Arabia		2,999 (8.1%)		2.6%	2.9%	1.8%	
Singapore		113 (0.3%)		2.6%	2.2%	3.7%	
South Africa		138 (0.4%)		2.6%	3.0%	1.5%	
Spain		730 (2%)		2.6%	2.7%	2.4%	
Uganda		320 (0.9%)		2.6%	1.8%	5.0%	
United Arab Emirates		1,718 (4.6%)		2.6%	2.9%	1.8%	
United Kingdom		205 (0.6%)		2.6%	2.5%	3.1%	
United States		271 (0.7%)		2.6%	2.7%	2.5%	

a Sample sizes of all participating countries differed. To control for over or underreporting from certain countries due to unequal survey collections, a survey weight created based on the country proportion in the total sample was applied in all analyses.

Valid percentage = responses only without considering missing values.

The corrected sentence appears below:

“Descriptive analyses, independent samples *t*-tests and chi-square tests (see Table 1) showed that scores of male and female respondents were different for all variables except for the perception of having more time.”

The second correction was made to the sentence that previously stated:

“To control for over or underreporting from certain countries due to unequal survey collections, a survey weight based on the country variable generated by SPSS for unbalanced samples was applied in all analyses.”

The corrected sentence appears below:

“To control for over or underreporting from certain countries due to unequal survey collections, a survey weight was created based on the country proportion in the total sample.”

A correction was made to the section **Results**, “*Participants*.” This sentence previously stated:

“A final *N* = 37,207 (77.8% women, *Mage* = 36.71, *SD* = 14.79) were retained for analysis.”

The corrected sentence appears below:

“A final *N* = 37,207 (73.6% women, *M*age = 36.72, *SD* = 14.43) were retained for analysis.”

Corrections have been made to the section **Results**, “*Descriptive Results*.” The paragraph previously stated:

“Mean scores for planning, selecting, and preparing healthier foods were average to high before the COVID-19 crisis in both women and men. All three food literacy behavior domains increased during the COVID-19 crisis in both women and men [plan, women, *F*_1,522, 232)_ = 25594.47, *p* < 0.01, men *F*_1,149, 036)_ = 2931.54, *p* < 0.01; select, women, *F*_1,522, 232)_ = 1088.85, *p* < 0.01, men *F*_1,149, 036)_ = 1153.84, *p* < 0.01; prepare, women, *F*_1,522, 232)_ = 9,819.70, *p* < 0.01, men *F*_1,149, 036)_ = 1054.81, *p* < 0.01, see Table 1 for all means and SD]. Furthermore, both men and women scored higher on financial stress when they had lost income due to COVID-19 [for women *t*_3,131, 242)_ = 296.81, *p* < 0.01 with *M* = 2.46, *SD* = 1.56 for women who did not lose income and *M* = 3.94, *SD* = 1.76 for women who lost income; for men *t*_3,131, 242)_ = 296.81, *p* < 0.01 with *M* = 2.46, *SD* = 1.58 for men who did not lose income and *M* = 4.04, *SD* = 1.79 for men who lost income].”

The corrected paragraph appears below:

“Mean scores for planning, selecting, and preparing healthier foods were average to high before the COVID-19 crisis in both women and men. All three food literacy behavior domains increased during the COVID-19 crisis in both women and men [plan, women, *t*_27,381)_ = 40.11, *p* < 0.001, men *t*_9,824)_ = 16.909, *p* < 0.001; select, women, *t*_27,381)_ = 3.25, *p* < 0.01, men *t*_9,824)_ = 8.63, *p* < 0.001; prepare, women, *t*_27,381)_ = 27.58, *p* < 0.001, men *t*_9,824)_ = 9.47, *p* < 0.001, see Table 1 for all means and *SD*]. Furthermore, both men and women scored higher on financial stress when they had lost income due to COVID-19 [for women *t*_15,092.38)_ = 71.87, *p* < 0.001 with *M* = 2.35, *SD* = 1.48 for women who did not lose income and *M* = 3.89, *SD* = 1.74 for women who lost income; for men *t*_7,005.57)_ = 45.05, *p* < 0.001 with *M* = 2.38, *SD* = 1.53 for men who did not lose income and *M* = 3.95, *SD* = 1.74 for men who lost income].”

Corrections have been made to the section **Results**, “*Hierarchical Multiple Regression Analyses*.” The first paragraph previously stated:

“Results of all hierarchical multiple regression analyses are reported in full detail in Supplementary Table 2, and summarized in Figures 1, 2 and 3. To start with the personal responses, the perception of having more time since the COVID-19 crisis was associated with increases in planning, selecting, and preparing healthier foods in both women and men (*p* < 0.01). COVID-19-induced financial stress was associated with decreases in planning and preparing healthier foods in both women and men (*p* < 0.01). Financial stress was further associated with an increased use of food labels and nutrition information among women (*p* < 0.01). COVID-19-induced psychological distress was associated with decreases in planning, selecting, and preparing healthier foods among women (*p* < 0.01). For men, psychological distress was negatively related to selecting–and positively related to preparing–healthier foods (*p* < 0.01).”

**Supplementary Table 2:** Detailed overview of all results from the Hierarchical Multiple Regression^a^ of the effects of COVID−19 induced personal and contextual factors on changes in planning. selecting. and preparing healthier foods (*N* = 37.207)^b^.

	**Women**								**Men**							
	***N*** **=27.013**								***N*** **=9.635**							
	**Model 1**				**Model 2**				**Model 1**				**Model 2**			
	**Personal factors controlled for socio–demographics**				**Personal and contextual factors controlled for socio–demographics**				**Personal factors controlled for socio–demographics**				**Personal and contextual factors controlled for socio–demographics**			
**Changes in planning**																
	**B**	**SE**	**Beta**	**sig**	**B**	**SE**	**Beta**	**sig**	**B**	**SE**	**Beta**	**sig**	**B**	**SE**	**Beta**	**sig**
Constant	0.343	0.045		< 0.001	0.208	0.049		< 0.001	0.162	0.069		0.018	0.144	0.073		0.048
*COVID−19 induced feelings*																
Financial stress	−0.028	0.004	−0.052	< 0.001	−0.028	0.004	−0.052	< 0.001	−0.025	0.007	−0.05	< 0.001	−0.026	0.007	−0.052	< 0.001
Feel they have more time	0.025	0.003	0.047	< 0.001	0.025	0.003	0.046	< 0.001	0.003	0.005	0.006	0.537	0.003	0.005	0.006	0.564
Kessler 6	−0.023	0.005	−0.031	< 0.001	−0.023	0.005	−0.032	< 0.001	0.008	0.008	0.012	0.319	0.008	0.008	0.012	0.321
*Social distancing measures*																
Forced to work from home					0.055	0.016	0.023	< 0.001					−0.024	0.024	−0.012	0.303
Public gatherings restricted					−0.037	0.015	−0.018	0.015					0.017	0.022	0.009	0.453
Private gatherings restricted					0.044	0.019	0.017	0.023					−0.034	0.028	−0.015	0.214
Restaurants closed					0.015	0.017	0.006	0.386					0.047	0.025	0.024	0.06
Bars/Pubs closed					0.03	0.018	0.013	0.087					−0.044	0.028	−0.021	0.11
Schools closed					0.061	0.017	0.023	< 0.001					0.051	0.025	0.023	0.043
*Sociodemographics – control variables*																
Age	0.001	0	0.013	0.084	0.001	0	0.012	0.127	0.001	0.001	0.013	0.318	0.001	0.001	0.012	0.341
Financial struggles	0	0.004	0	0.951	−0.001	0.004	−0.001	0.897	−0.015	0.007	−0.03	0.032	−0.014	0.007	−0.028	0.046
Financial struggles for food	−0.028	0.004	−0.053	< 0.001	−0.025	0.004	−0.048	< 0.001	−0.013	0.006	−0.027	0.044	−0.012	0.006	−0.026	0.054
Loss of income	−0.024	0.013	−0.012	0.071	−0.023	0.013	−0.011	0.086	−0.014	0.02	−0.008	0.495	−0.013	0.02	−0.007	0.528
Highest obtained degree	0.032	0.006	0.033	< 0.001	0.028	0.006	0.029	< 0.001	0.032	0.009	0.039	< 0.001	0.03	0.009	0.037	< 0.001
Employment status	−0.015	0.009	−0.011	0.109	−0.011	0.009	−0.008	0.228	0.024	0.016	0.018	0.14	0.026	0.016	0.019	0.114
Number of cohabiting adults	−0.008	0.003	−0.017	0.012	−0.007	0.003	−0.015	0.03	−0.009	0.005	−0.018	0.106	−0.01	0.005	−0.02	0.068
Number of cohabiting children	−0.039	0.004	−0.057	< 0.001	−0.036	0.004	−0.053	< 0.001	−0.036	0.007	−0.055	< 0.001	−0.037	0.007	−0.057	< 0.001
	*F (11, 490505) = 52.635. p < 0.001*				*F (17, 539337) = 37.52. p < 0.001*				*F (11, 11594) = 14.07. p < 0.001*				*F (17, 123872) = 9.732. p < 0.001*			
*Adjusted R^2^*	0.021				0.022				0.015				0.015			
*Change R^2^*	0.021 p < 0.001				0.002 p < 0.001				0.016 p < 0.001				0.001 p =0.102			
																
**Changes in Selecting**																
	**B**	**SE**	**Beta**	**sig**	**B**	**SE**	**Beta**	**sig**	**B**	**SE**	**Beta**	**sig**	**B**	**SE**	**Beta**	**sig**
Constant	−0.056	0.056		0.321	0.04	0.061		0.519	−0.021	0.084		0.803	0.063	0.09		0.481
*COVID-19 induced feelings*																
Financial Stress	0.008	0.005	0.012	0.128	0.006	0.005	0.009	0.286	0	0.008	0.001	0.954	−0.002	0.008	−0.003	0.824
Feel to have more time	0.024	0.004	0.037	< 0.001	0.024	0.004	0.037	< 0.001	0.011	0.006	0.018	0.083	0.012	0.006	0.019	0.076
Kessler 6	−0.057	0.006	−0.062	< 0.001	−0.058	0.006	−0.063	< 0.001	−0.023	0.01	−0.027	0.026	−0.025	0.01	−0.03	0.013
*Social Distancing Measures*																
Forced to work from home					−0.063	0.02	−0.021	0.002					−0.031	0.029	−0.012	0.283
Public gatherings restricted					0.079	0.019	0.03	< 0.001					0.049	0.028	0.021	0.077
Private gatherings restricted					0.004	0.024	0.001	0.872					0.034	0.034	0.012	0.314
Restaurants closed					−0.034	0.021	−0.012	0.106					−0.042	0.031	−0.017	0.175
Bars/Pubs closed					−0.073	0.022	−0.025	< 0.001					−0.063	0.034	−0.024	0.066
Schools closed					0.053	0.021	0.016	0.011					0.018	0.031	0.007	0.564
*Sociodemographics – control variables*																
Age	−0.002	0.001	−0.021	0.005	−0.002	0.001	−0.02	0.01	0.001	0.001	0.018	0.155	0.001	0.001	0.019	0.135
Financial struggles	0.009	0.005	0.014	0.078	0.011	0.005	0.016	0.046	−0.006	0.008	−0.009	0.501	−0.003	0.008	−0.006	0.688
Financial struggles for food	0.014	0.005	0.021	0.005	0.011	0.005	0.017	0.025	0.011	0.008	0.02	0.142	0.01	0.008	0.017	0.217
Loss of income	0.066	0.017	0.027	< 0.001	0.068	0.017	0.028	< 0.001	0.013	0.025	0.006	0.594	0.016	0.025	0.007	0.527
Highest obtained degree	−0.018	0.008	−0.015	0.017	−0.016	0.008	−0.013	0.045	−0.012	0.011	−0.012	0.269	−0.012	0.011	−0.012	0.287
Employment status	0.024	0.011	0.015	0.038	0.018	0.011	0.011	0.124	0.037	0.02	0.022	0.068	0.034	0.02	0.02	0.089
Number of cohabiting adults	0.012	0.004	0.02	0.002	0.008	0.004	0.013	0.05	0.013	0.007	0.022	0.046	0.012	0.007	0.019	0.08
Number of cohabiting children	0.004	0.005	0.005	0.414	−0.002	0.006	−0.002	0.705	−0.019	0.009	−0.024	0.026	−0.023	0.009	−0.029	0.008
	*F (11,241205) = 16.772. p < 0.001*				*F (17,329201) = 14.845. p < 0.001*				*F (11,3494) = 2.801. p = .001*				*F (17,56146) = 2.916. p < 0.001*			
*Adjusted R^2^*	0.006				0.009				0.002				0.003			
*Change R^2^*	0.007 p < 0.001				0.002 p < 0.001				0.003 p =.001				0.002 p = .005			
																
**Changes in preparation**																
	**B**	**SE**	**Beta**	**sig**	**B**	**SE**	**Beta**	**sig**	**B**	**SE**	**Beta**	**sig**	**B**	**SE**	**Beta**	**sig**
Constant	0.251	0.047		< 0.001	0.128	0.051		0.012	0.19	0.073		0.009	0.216	0.078		0.005
*COVID-19 induced feelings*																
Financial Stress	−0.027	0.005	−0.049	< 0.001	−0.027	0.005	−0.049	< 0.001	−0.031	0.007	−0.059	< 0.001	−0.031	0.007	−0.06	< 0.001
Feel they have more time	0.038	0.003	0.069	< 0.001	0.038	0.003	0.069	< 0.001	0.01	0.006	0.018	0.087	0.01	0.006	0.018	0.085
Kessler 6	−0.013	0.005	−0.017	0.011	−0.013	0.005	−0.017	0.013	0.017	0.009	0.022	0.062	0.016	0.009	0.022	0.071
*Social distancing measures*																
Forced to work from home					0.08	0.017	0.06	< 0.001					−0.038	0.025	−0.017	0.137
Public gatherings restricted					−0.02	0.016	−0.009	0.209					0.001	0.024	0	0.973
Private gatherings restricted					0.015	0.02	0.006	0.44					−0.067	0.029	−0.027	0.023
Restaurants closed					0.002	0.017	0.001	0.93					0.001	0.026	0	0.975
Bars/Pubs closed					0.024	0.018	0.01	0.193					−0.019	0.03	−0.008	0.515
Schools closed					0.037	0.018	0.013	0.036					0.032	0.027	0.014	0.232
*Sociodemographics – control variables*																
Age	−0.001	0.001	−0.021	0.005	−0.002	0.001	−0.023	0.003	−0.001	0.001	−0.015	0.23	−0.001	0.001	−0.013	0.305
Financial struggles	0.004	0.004	0.008	0.326	0.004	0.004	0.007	0.367	−0.005	0.007	−0.01	0.477	−0.004	0.007	−0.008	0.55
Financial struggles for food	−0.023	0.004	−0.043	< 0.001	−0.021	0.004	−0.038	< 0.001	−0.008	0.007	−0.016	0.225	−0.008	0.007	−0.016	0.218
Loss of income	−0.006	0.014	−0.003	0.675	−0.005	0.014	−0.003	0.706	−0.008	0.022	−0.004	0.712	−0.007	0.022	−0.004	0.741
Highest obtained degree	0.028	0.006	0.027	< 0.001	0.024	0.006	0.024	< 0.001	0.037	0.009	0.043	< 0.001	0.036	0.009	0.042	< 0.001
Employment status	−0.026	0.01	−0.019	0.006	−0.022	0.01	−0.017	0.019	−0.03	0.017	−0.02	0.089	−0.03	0.017	−0.02	0.09
Number of cohabiting adults	−0.008	0.003	−0.016	0.017	−0.007	0.003	−0.013	0.049	−0.012	0.006	−0.023	0.033	−0.013	0.006	−0.024	0.03
Number of cohabiting children	−0.036	0.005	−0.051	< 0.001	−0.033	0.005	−0.046	< 0.001	−0.044	0.008	−0.062	< 0.001	−0.045	0.008	−0.064	< 0.001
	*F (11,400952) = 40.002. p < 0.001*				*F (17,441845) = 28.565. p < 0.001*				*F (11,111938) = 12.009. p < 0.001*				*F (17,122019) = 8.476. p < 0.001*			
*Adjusted R^2^*	0.016				0.017				0.012				0.013			
*Change R^2^*	0.016 p < 0.001				0.002 p < 0.001				0.014 p < 0.001				0.001 p = .064			

a Separate regressions were used for planning. selecting. and preparing healthier foods for male and female participants. In a first step only personal factors were included. in a second step social distancing measures were added to the model. In both models we controlled for a range of sociodemographic variables known to relate to food literacy. We report the unstandardized beta (B). standard error for the unstandardized beta (SE) and the standardized beta.

b Sample sizes off all participating countries differed. To control for over or underreporting from certain countries due to unequal survey collections. a survey weight created based on the country proportion in the total sample was applied in all analyses.

**Figure 1:**
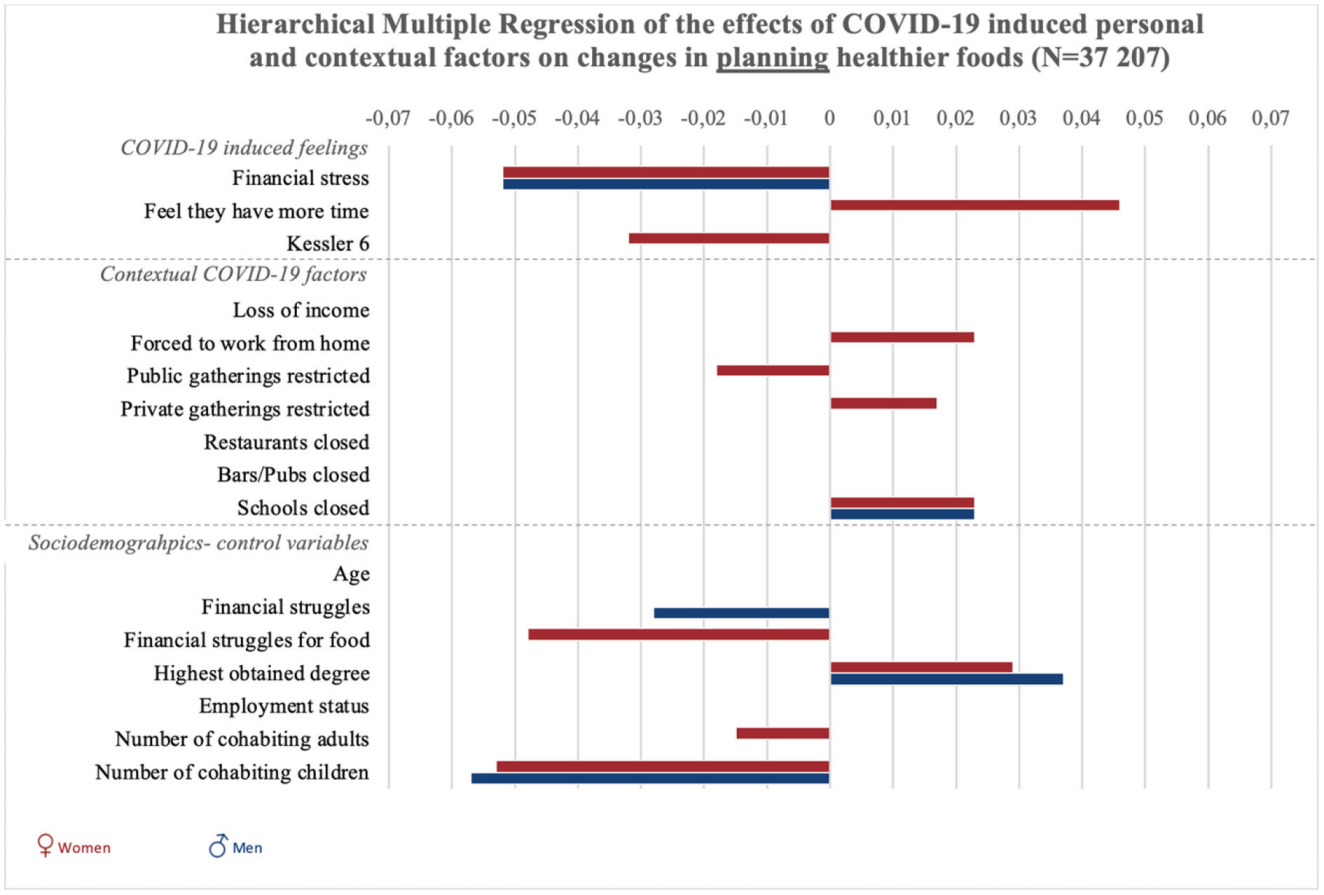
Graphic summary of the significant relations between personal, contextual and sociodemographic variables and changes in planning healthier foods during COVID-19. We report beta-values only for significant relations in models 2 for planning healthier foods. Bars to the right indicate improvement in food planning, bars to the left indicate decreases in planning healthy foods.

**Figure 2:**
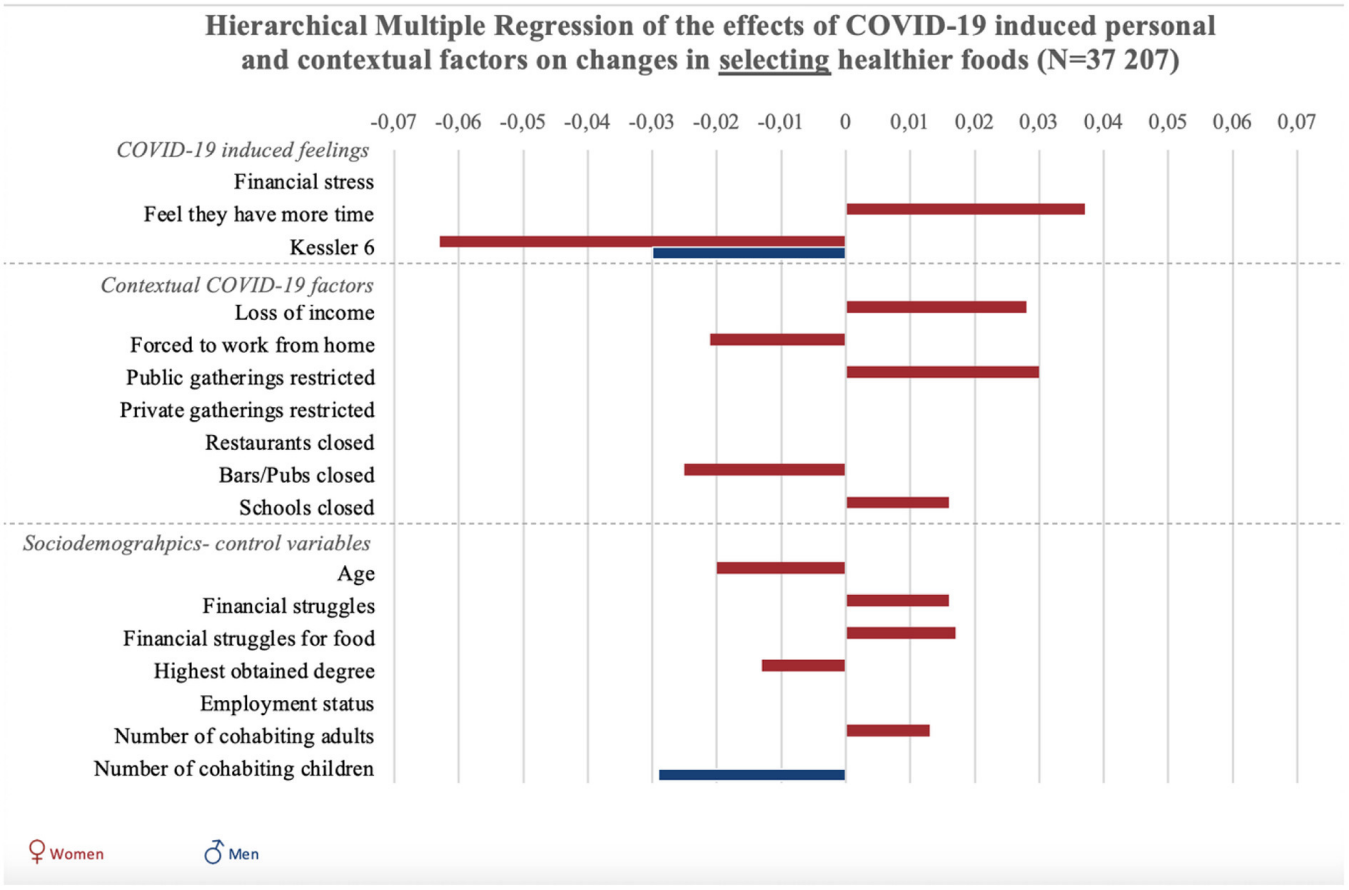
Graphic summary of the significant relations between personal, contextual and sociodemographic variables and changes in selecting healthier foods during COVID-19. We report beta-values only for significant relations in models 2 for selecting healthier foods. Bars to the right indicate improvement in food selection, bars to the left indicate decreases in selecting healthy foods.

**Figure 3:**
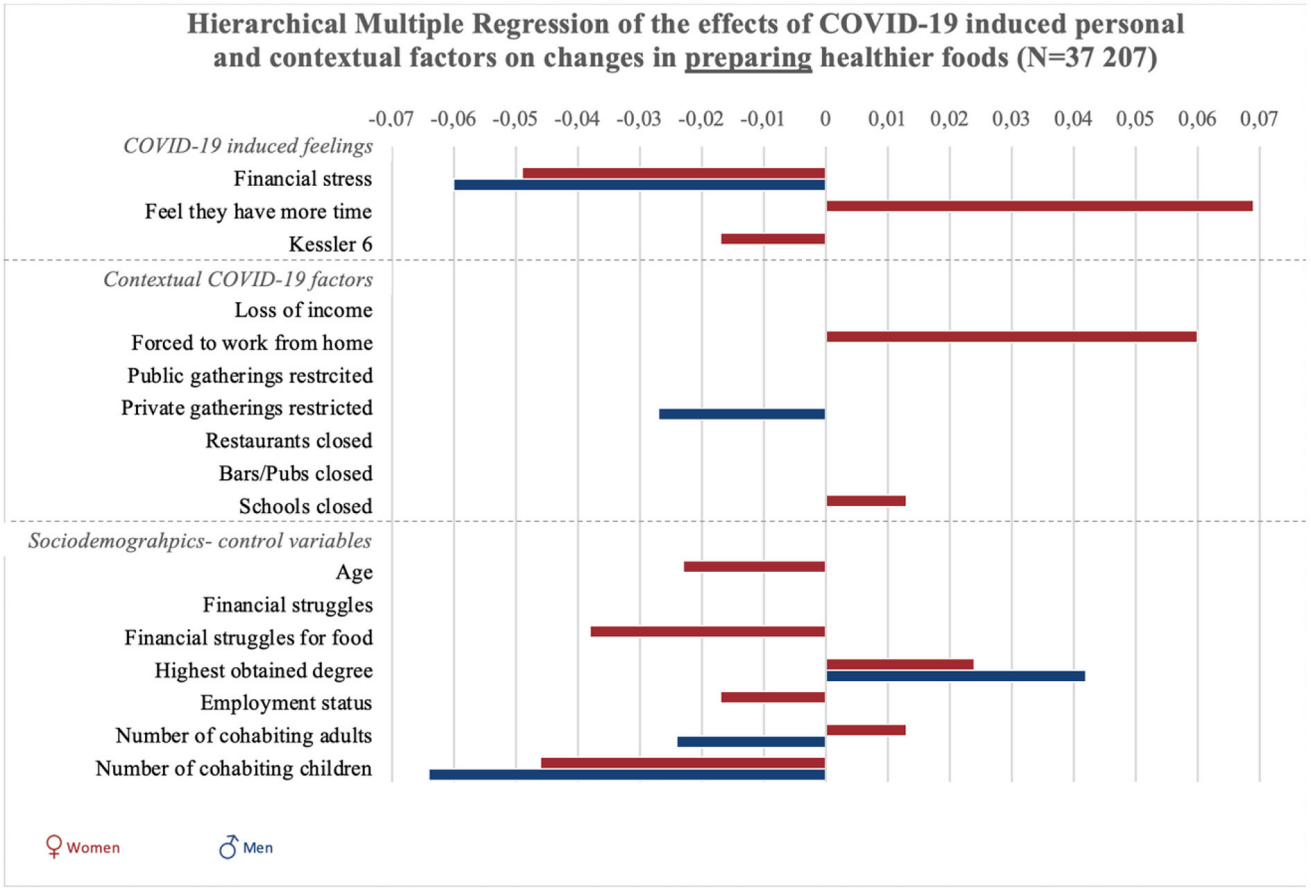
Graphic summary of the significant relations between personal, contextual and sociodemographic variables and changes in preparing healthier foods during COVID-19. We report beta-values only for significant relations in models 2 for preparing healthier foods. Bars to the right indicate improvement in food preparation, bars to the left indicate decreases in preparing healthy foods.

The corrected first paragraph appears below:

“Results of all hierarchical multiple regression analyses are reported in full detail in Supplementary Table 2, and summarized in Figures 1, 2 and 3. To start with the personal responses, the perception of having more time since the COVID-19 crisis was associated with increases in planning, selecting, and preparing healthier foods in women (*p* < 0.001), but not significantly in men (*p* = 0.54). COVID-19-induced financial stress was associated with decreases in planning and preparing healthier foods in both women and men (*p* < 0.001). COVID-19-induced psychological distress was associated with decreases in planning, selecting, and preparing healthier foods among women (*p* < 0.05). For men, psychological distress was negatively related to selecting healthier foods (*p* < 0.05).”

The second paragraph previously stated:

“Concerning contextual factors, positive associations were found between policies to stay at home/work from home and changes in planning and preparing healthier foods in both women and men (*p* < 0.01). However, staying home was negatively associated with selecting healthier foods in women and men (*p* < 0.01). Next, policies on public gatherings related to an increase in selecting healthier foods among women, but this association was negative for men (*p* < 0.01). Policies on public gatherings also negatively related to women's planning and preparing of healthier foods. Policies on private gatherings negatively related to men's planning and preparation of healthier foods (*p* < 0.01).”

The corrected second paragraph appears below:

“Concerning contextual factors, positive associations were found between policies to stay at home/work from home and changes in planning and preparing healthier foods in women (*p* < 0.001). However, staying home was negatively associated with selecting healthier foods in women (*p* < 0.01). Next, policies on public gatherings related to an increase in selecting healthier foods among women (*p* < 0.01). Policies on public gatherings also negatively related to women's planning of healthier foods (*p* < 0.05). Policies on private gatherings positively related to women's planning (*p* < 0.05), and was negatively related to men's preparation of healthier foods (*p* < 0.05).”

The third paragraph previously stated:

“The closure of schools was associated with increased healthier food selection in men and women (*p* < 0.01), but decreased healthier food planning in men and preparation in women (*p* < 0.01). The closure of restaurants and the closure of pubs and bars was associated with decreases in selecting healthier foods in men and women (*p* < 0.01). The closure of restaurants, pubs, and bars further increased women's healthier food planning, while healthier food planning decreased in men when pubs/bars were closed (*p* < 0.01). And while women's preparation of healthier meals increased when restaurants were closed, men reported that their preparation of healthier meals decreased (*p* < 0.01).”

The corrected third paragraph appears below:

“The closure of schools was associated with increased healthier food planning in men and women, as well as selection and preparation in women (*p* < 0.05).The closure of pubs and bars was associated with decreases in selecting healthier foods in women (*p* < 0.001).”

The fourth paragraph previously stated:

“Regarding the sociodemographic characteristics associated with changes in food literacy behaviors, educational attainment was negatively related to changes in selecting healthier foods and positively related to changes in planning and preparing healthier foods in men and women (*p* < 0.01). Employment status was negatively related to changes in food preparation in men and women (*p* < 0.01) and positively related to changes in selecting healthier foods in women. Struggling to make money last until the next payday was positively related to changes in women's selecting healthier foods (*p* < 0.01), and negatively related to men's changes in food planning (*p* < 0.01). Struggling to have enough money to go shopping for food was also related to positive changes in women's use of food labels (selecting healthier foods), but related to negative changes in both women and men's planning and preparing healthier foods (*p* < 0.01). Also loss of income was related to an increase in selecting healthier foods among women and men (*p* < 0.01), an increase in preparing healthier meals in women, and a decrease in preparing healthier meals in men (*p* < 0.01). Age was positively related to changes in planning healthier foods for men and women. It was also positively related to changes in men's healthier food selection, while for women it was negatively related to changes in selecting and preparing healthier foods (*p* < 0.01). Finally, the more adult cohabitants women had during the COVID-19 crisis, the more their selection and preparation of healthier foods improved (*p* < 0.01). For men, increases in the number of adult cohabitants related to decreases in planning and preparing healthier foods (*p* < 0.01). The number of children in the household was negatively associated with men and women's planning and preparation of healthier foods (*p* < 0.01), and positively associated with women's selection of healthier foods.”

The corrected fourth paragraph appears below:

“Regarding the sociodemographic characteristics associated with changes in food literacy behaviors, educational attainment was negatively related to changes in selecting healthier foods in women (*p* < 0.05) and positively related to changes in planning and preparing healthier foods in men and women (*p* < 0.001). Employment status was negatively related to changes in food preparation in women (*p* < 0.05). Struggling to make money last until the next payday was positively related to changes in women's selecting healthier foods (*p* < 0.05), and negatively related to men's changes in food planning (*p* < 0.05). Struggling to have enough money to go shopping for food was also related to positive changes in women's use of food labels (selecting healthier foods), but related to negative changes in women's planning and preparing healthier foods (*p* < 0.01). Also loss of income was related to an increase in selecting healthier foods among women (*p* < 0.001). For women, age was negatively related to changes in selecting and preparing healthier foods (*p* < 0.01). Finally, the more adult cohabitants men had during the COVID-19 crisis, the more their preparation of healthier foods decreased (*p* < 0.01). For women, increases in the number of adult cohabitants related to decreases in planning healthier foods (*p* < 0.05). The number of children in the household was negatively associated with men and women's planning and preparation of healthier foods (*p* < 0.001), and also negatively associated with men's selection of healthier foods (*p* < 0.01).”

Corrections have been made to the section **Discussion**. The second paragraph previously stated:

“First, the COVID-19 crisis has taught us that stay-at-home policies, and especially personal perceptions of having more time, can increase the willingness to plan, select, and prepare healthier foods. Stay-at-home policies resulted in distorted perceptions of time and made many people feel bored (12, 13). Yet, stay-at-home policies may be in our favor when it comes to food literacy, if people feel to have more time, because in these cases we observed positive increases in planning, preparing, and selecting healthier foods. A health equity lens is warranted (3), however, since working from home is not beneficial for everyone and can lead to increased stress in some people (20). Results also show that while feeling to have more time relates to increases in planning, selecting and preparing healthier foods, stay-at-home policies corresponded to decreases in selecting healthier foods as well. Moreover, women with young children in particular experience more stress and time constraints when working from home (22). We also observed that an increase in the number of children one lives with relates to a decrease in changes in planning and preparing healthier foods. Thus, health practitioners should find ways of incorporating workplace policies to increase time availability in long-term food literacy interventions, bearing the home situation in mind. The requirement to work from home has been a successful public health initiative to curb the spread of COVID-19, and may be a successful long-term strategy to improve food literacy, other factors considered.”

The corrected second paragraph appears below:

“First, the COVID-19 crisis has taught us that stay-at-home policies, and especially personal perceptions of having more time among women, can increase the willingness to plan, select, and prepare healthier foods. Stay-at-home policies resulted in distorted perceptions of time and made many people feel bored (12, 13). Yet, stay-at-home policies may be in our favor when it comes to food literacy, if people feel to have more time, because in these cases we observed positive increases in planning, preparing, and selecting healthier foods among women. A health equity lens is warranted (3), however, since working from home is not beneficial for everyone and can lead to increased stress in some people (20). This is reflected in our results showing that while feeling to have more time relates to increases in planning, selecting, and preparing healthier foods among women, stay-at-home policies corresponded to decreases in selecting healthier foods as well among this group. These seemingly contradicting results can perhaps be brought back to time perception, as time constraints are an important factor in practicing healthy food behaviors (21). Stay-at-home policies specifically could be responsible for this dual outcome of either experiencing more or less time constraints, as some have experienced having more time during COVID-19 work from home obligations (13), and others—mainly parents and mothers especially—have had less or more fragmented time perceptions (22). Mothers during COVID-19 have especially perceived more time-related stress in combing their work and home responsibilities (22), aligning with previous findings that women with young children in particular experience more stress and time constraints when working from home (23). We also observed that an increase in the number of children one lives with relates to a decrease in changes in planning and preparing healthier foods in men and women, as well as selecting them for men. Thus, health practitioners should find ways of incorporating workplace policies to increase time availability in long-term food literacy interventions, bearing the home situation in mind for parents and especially mothers. The requirement to work from home has been a successful public health initiative to curb the spread of COVID-19, and may be a successful long-term strategy to improve food literacy, other factors considered.”

The third paragraph, from the third sentence, previously stated:

“Idyllic representations of relieving stress in the kitchen during the COVID-19 crisis (2) may not have applied to women in our study. Among men we did observe an increase in preparing healthier meals when psychological distress increased. This could be interpreted as men viewing cooking as a “leisure” activity (22), while women take up the “burden” of everyday cooking (23). This may explain why, during the COVID-19 crisis, psychological distress became a barrier to women's everyday cooking but a creative outlet for men as a way to relieve stress (16). Given that women are more likely to be responsible for everyday food preparation in households, the negative impact of psychological distress on their food literacy behaviors may impact the health of many other children and adults.”

The corrected third paragraph, from the third sentence, appears below:

“Increases in psychological distress have been linked to averse nutritional health behaviors in the past (24). Previous studies have highlighted different possible causes to increased distress as a result of COVID-19 lockdown. Some studies have cited the distorted time perceptions and a sense of timelessness as a possible cause for sadness psychological distress (12, 13). Others cite lower socioeconomic status, COVID-19 infection risk, and longer media exposure as factors related to psychological distress (25). Women especially have been associated with higher psychological distress (25), which could explain our findings as they related to food literacy behaviors.”

The fourth paragraph, from the third till the seventh sentence, previously stated:

“Both loss of income and feelings of financial stress caused by the COVID-19 crisis, as well as struggling to have enough money for food related to increases in selecting healthier foods for women. When looking at the planning and preparation of healthier meals, however, results show a different pattern: financial stress and struggles to have enough money for food related to decreases in planning and preparing healthier meals. Thus, while financial stress and - constraints do not relate to women's planning and preparation of healthier meals, something did change in their food shopping behavior. A potential explanation for this may be that prices of certain foods became more expensive, especially for foods that were hoarded due to social panic (24).”

The corrected fourth paragraph, from the third till the seventh sentence, appears below:

“Loss of income and struggling to have enough money for food related to increases in selecting healthier foods for women. When looking at the planning and preparation of healthier meals, however, results show a different pattern: financial stress related to decreases in planning and preparing healthier meals for both men and women, whereas struggles to have enough money for food related to these decreases only among women. Thus, while financial stress and -constraints decreased women's planning and preparation of healthier meals, it seemed to increase their selection of healthy meals. A potential explanation for this may be found in grocery shopping as it relates to meal selection, as prices of certain foods became more expensive, especially for foods that were hoarded due to social panic (26).”

The fifth paragraph previously stated:

“With regard to other sociodemographic characteristics, our results show that increases in food planning were associated with older age in men and women, while for women age was related negatively to changes in selecting and preparing healthier foods. A potential explanation for this is that more women acquire higher levels of food literacy at a younger age than men, leaving less room for improvement as they get older (4, 5, 7, 10).”

The corrected fifth paragraph appears below:

“With regard to other sociodemographic characteristics, our results show that increases in food planning were associated with older age in men and women, while, for women, age was related negatively to changes in selecting and preparing healthier foods. A potential explanation for this is that more women acquire higher levels of food literacy at a younger age than men, leaving less room for improvement as they get older (4, 5, 7, 10). Additionally, these results can be linked to younger age being associated with increased psychological distress during COVID-19 (25), potentially causing less healthy food behaviors (24).”

The eighth paragraph previously stated:

“In conclusion, we reported overall increases in planning, selecting, and preparing healthier foods during the COVID-19 crisis among women and men in 38 countries around the world using self-report data. Perceptions of having more time were most clearly associated with these positive changes, followed by the contextual factor of stay-at-home policies. Psychological distress was related to decreases in women's food literacy, and increases in men's healthy food preparation. Financial stress was not always related to decreases in food literacy; especially among women, financial stress and struggles related to increased healthier food selection behaviors.”

The eight paragraph appears below:

“In conclusion, we reported overall increases in planning, selecting, and preparing healthier foods during the COVID-19 crisis among women and men in 38 countries around the world using self-report data. Perceptions of having more time were most clearly associated with these positive changes among women, followed by the contextual factor of stay-at-home policies. Psychological distress was related to decreases in women's food literacy, and decreases in men's healthy food selection. Financial stress was not always related to decreases in food literacy, financial stress and struggles related to increased healthier food selection behaviors among women but decreased in planning and preparing.”

In the original article, there was an error in Figure 1 as published. An incorrect weighting coefficient was used, therefore analyses where ran again using the correct weighting variable. The corrected Figure 1 and its caption appear below.

In the original article, there was an error in Figure 2 as published. An incorrect weighting coefficient was used, therefore analyses where ran again using the correct weighting variable. The corrected Figure 2 and its caption appear below.

In the original article, there was an error in Figure 3 as published. An incorrect weighting coefficient was used, therefore analyses where ran again using the correct weighting variable. The corrected Figure 3 and its caption appear below.

In the original article, there was an error in Table 1 as published. An incorrect weighting coefficient was used, therefore analyses where ran again using the correct weighting variable. The corrected Table 1 and its caption appear below.

In the original article, there was an error in Supplementary Table 2 as published. An incorrect weighting coefficient was used, therefore analyses where ran again using the correct weighting variable. Supplementary Table 2 and its caption has been updated in the original article.

**Additional correction to text (Materials and Methods)**

In the original article, it was stated that repeated measures ANOVA was used to test the significance of changes. However, the reported analyses were paired-samples *t*-tests. Therefore, a correction was made to **Materials and Methods**, “*Study Size and Statistical Analysis*,” paragraph 1. The sentence previously stated:

“Repeated measures ANOVA was first used to test the significance of changes in self-reported planning, selection, and preparation of healthier foods before vs. during COVID-19.”

The corrected sentence appears below:

“Paired-samples *t*-test was first used to test the significance of changes in self-reported planning, selection, and preparation of healthier foods before vs. during COVID-19.”

The authors apologize for these errors and state that the key message of the publication remains intact. The original article has been updated.

## Publisher's note

All claims expressed in this article are solely those of the authors and do not necessarily represent those of their affiliated organizations, or those of the publisher, the editors and the reviewers. Any product that may be evaluated in this article, or claim that may be made by its manufacturer, is not guaranteed or endorsed by the publisher.
