# Supplementary material for: An Evaluation of the COVID-19 Pandemic and Perceived Social Distancing Policies in Relation to Planning, Selecting, and Preparing Healthy Meals: An Observational Study in 38 Countries Worldwide
Source: Front Nutr. 2021 Feb 4;7:621726. doi: 10.3389/fnut.2020.621726 (PMC7890074; doi:10.3389/fnut.2020.621726)
Supplement: Supplementary file 1 [file Table_1.DOCX]

Table 2. Detailed overview of all results from the Hierarchical Multiple Regression^a^ of the effects of COVID-19 induced personal and contextual factors on changes in planning, selecting, and preparing healthier foods (N=37 207)^b^.

|  | **Women** | | | | | | | | **Men** | | | | | | | |
| --- | --- | --- | --- | --- | --- | --- | --- | --- | --- | --- | --- | --- | --- | --- | --- | --- |
|  | **N=27 013** | | | | | | | | **N=9635** | | | | | | | |
|  | **Model 1** | | | | **Model 2** | | | | **Model 1** | | | | **Model 2** | | | |
|  | **Personal factors controlled for socio-demographics** | | | | **Personal & contextual factors controlled for socio-demographics** | | | | **Personal factors controlled for socio-demographics** | | | | **Personal & contextual factors controlled for socio-demographics** | | | |
| **Changes in Planning** |  |  |  |  |  |  |  |  |  |  |  |  |  |  |  |  |
|  | **B** | **SE** | **Beta** | **sig** | **B** | **SE** | **Beta** | **sig** | **B** | **SE** | **Beta** | **sig** | **B** | **SE** | **Beta** | **sig** |
| Constant | 0,343 | 0,045 |  | <.001 | 0,208 | 0,049 |  | <.001 | 0,162 | 0,069 |  | 0,018 | 0,144 | 0,073 |  | 0,048 |
| *COVID-19 induced feelings* |  |  |  |  |  |  |  |  |  |  |  |  |  |  |  |  |
| Financial Stress | -0,028 | 0,004 | -0,052 | <.001 | -0,028 | 0,004 | -0,052 | <.001 | -0,025 | 0,007 | -0,05 | <.001 | -0,026 | 0,007 | -0,052 | <.001 |
| Feel they have more time | 0,025 | 0,003 | 0,047 | <.001 | 0,025 | 0,003 | 0,046 | <.001 | 0,003 | 0,005 | 0,006 | 0,537 | 0,003 | 0,005 | 0,006 | 0,564 |
| Kessler 6 | -0,023 | 0,005 | -0,031 | <.001 | -0,023 | 0,005 | -0,032 | <.001 | 0,008 | 0,008 | 0,012 | 0,319 | 0,008 | 0,008 | 0,012 | 0,321 |
| *Social Distancing Measures* |  |  |  |  |  |  |  |  |  |  |  |  |  |  |  |  |
| Forced to work from home |  |  |  |  | 0,055 | 0,016 | 0,023 | <.001 |  |  |  |  | -0,024 | 0,024 | -0,012 | 0,303 |
| Public gatherings restricted |  |  |  |  | -0,037 | 0,015 | -0,018 | 0,015 |  |  |  |  | 0,017 | 0,022 | 0,009 | 0,453 |
| Private gatherings restricted |  |  |  |  | 0,044 | 0,019 | 0,017 | 0,023 |  |  |  |  | -0,034 | 0,028 | -0,015 | 0,214 |
| Restaurants closed |  |  |  |  | 0,015 | 0,017 | 0,006 | 0,386 |  |  |  |  | 0,047 | 0,025 | 0,024 | 0,06 |
| Bars/Pubs closed |  |  |  |  | 0,03 | 0,018 | 0,013 | 0,087 |  |  |  |  | -0,044 | 0,028 | -0,021 | 0,11 |
| Schools closed |  |  |  |  | 0,061 | 0,017 | 0,023 | <.001 |  |  |  |  | 0,051 | 0,025 | 0,023 | 0,043 |
| *Sociodemographics – control variables* |  |  |  |  |  |  |  |  |  |  |  |  |  |  |  |  |
| Age | 0,001 | 0 | 0,013 | 0,084 | 0,001 | 0 | 0,012 | 0,127 | 0,001 | 0,001 | 0,013 | 0,318 | 0,001 | 0,001 | 0,012 | 0,341 |
| Financial struggles | 0 | 0,004 | 0 | 0,951 | -0,001 | 0,004 | -0,001 | 0,897 | -0,015 | 0,007 | -0,03 | 0,032 | -0,014 | 0,007 | -0,028 | 0,046 |
| Financial struggles for food | -0,028 | 0,004 | -0,053 | <.001 | -0,025 | 0,004 | -0,048 | <.001 | -0,013 | 0,006 | -0,027 | 0,044 | -0,012 | 0,006 | -0,026 | 0,054 |
| Loss of income | -0,024 | 0,013 | -0,012 | 0,071 | -0,023 | 0,013 | -0,011 | 0,086 | -0,014 | 0,02 | -0,008 | 0,495 | -0,013 | 0,02 | -0,007 | 0,528 |
| Highest obtained degree | 0,032 | 0,006 | 0,033 | <.001 | 0,028 | 0,006 | 0,029 | <.001 | 0,032 | 0,009 | 0,039 | <.001 | 0,03 | 0,009 | 0,037 | <.001 |
| Employment status | -0,015 | 0,009 | -0,011 | 0,109 | -0,011 | 0,009 | -0,008 | 0,228 | 0,024 | 0,016 | 0,018 | 0,14 | 0,026 | 0,016 | 0,019 | 0,114 |
| Number of cohabiting adults | -0,008 | 0,003 | -0,017 | 0,012 | -0,007 | 0,003 | -0,015 | 0,03 | -0,009 | 0,005 | -0,018 | 0,106 | -0,01 | 0,005 | -0,02 | 0,068 |
| Number of cohabiting children | -0,039 | 0,004 | -0,057 | <.001 | -0,036 | 0,004 | -0,053 | <.001 | -0,036 | 0,007 | -0,055 | <.001 | -0,037 | 0,007 | -0,057 | <.001 |
|  | *F (11, 490.505) = 52.635, p <.001* | | | | *F (17, 539.337) = 37.52, p <.001* | | | | *F (11, 115.94) = 14.07, p <.001* | | | | *F (17, 123.872) = 9.732, p <.001* | | | |
| *Adjusted R^2^* | 0,021 |  |  |  | 0,022 |  |  |  | 0,015 |  |  |  | 0,015 |  |  |  |
| *Change R^2^* | 0,021 | p <.001 |  |  | 0,002 | p <.001 |  |  | 0,016 | p <.001 |  |  | 0,001 | p =0.102 |  |  |
|  |  |  |  |  |  |  |  |  |  |  |  |  |  |  |  |  |
| **Changes in Selecting** |  |  |  |  |  |  |  |  |  |  |  |  |  |  |  |  |
|  | **B** | **SE** | **Beta** | **sig** | **B** | **SE** | **Beta** | **sig** | **B** | **SE** | **Beta** | **sig** | **B** | **SE** | **Beta** | **sig** |
| Constant | -0,056 | 0,056 |  | 0,321 | 0,04 | 0,061 |  | 0,519 | -0,021 | 0,084 |  | 0,803 | 0,063 | 0,09 |  | 0,481 |
| *COVID-19 induced feelings* |  |  |  |  |  |  |  |  |  |  |  |  |  |  |  |  |
| Financial Stress | 0,008 | 0,005 | 0,012 | 0,128 | 0,006 | 0,005 | 0,009 | 0,286 | 0 | 0,008 | 0,001 | 0,954 | -0,002 | 0,008 | -0,003 | 0,824 |
| Feel to have more time | 0,024 | 0,004 | 0,037 | <.001 | 0,024 | 0,004 | 0,037 | <.001 | 0,011 | 0,006 | 0,018 | 0,083 | 0,012 | 0,006 | 0,019 | 0,076 |
| Kessler 6 | -0,057 | 0,006 | -0,062 | <.001 | -0,058 | 0,006 | -0,063 | <.001 | -0,023 | 0,01 | -0,027 | 0,026 | -0,025 | 0,01 | -0,03 | 0,013 |
| *Social Distancing Measures* |  |  |  |  |  |  |  |  |  |  |  |  |  |  |  |  |
| Forced to work from home |  |  |  |  | -0,063 | 0,02 | -0,021 | 0,002 |  |  |  |  | -0,031 | 0,029 | -0,012 | 0,283 |
| Public gatherings restricted |  |  |  |  | 0,079 | 0,019 | 0,03 | <.001 |  |  |  |  | 0,049 | 0,028 | 0,021 | 0,077 |
| Private gatherings restricted |  |  |  |  | 0,004 | 0,024 | 0,001 | 0,872 |  |  |  |  | 0,034 | 0,034 | 0,012 | 0,314 |
| Restaurants closed |  |  |  |  | -0,034 | 0,021 | -0,012 | 0,106 |  |  |  |  | -0,042 | 0,031 | -0,017 | 0,175 |
| Bars/Pubs closed |  |  |  |  | -0,073 | 0,022 | -0,025 | <.001 |  |  |  |  | -0,063 | 0,034 | -0,024 | 0,066 |
| Schools closed |  |  |  |  | 0,053 | 0,021 | 0,016 | 0,011 |  |  |  |  | 0,018 | 0,031 | 0,007 | 0,564 |
| *Sociodemographics – control variables* |  |  |  |  |  |  |  |  |  |  |  |  |  |  |  |  |
| Age | -0,002 | 0,001 | -0,021 | 0,005 | -0,002 | 0,001 | -0,02 | 0,01 | 0,001 | 0,001 | 0,018 | 0,155 | 0,001 | 0,001 | 0,019 | 0,135 |
| Financial struggles | 0,009 | 0,005 | 0,014 | 0,078 | 0,011 | 0,005 | 0,016 | 0,046 | -0,006 | 0,008 | -0,009 | 0,501 | -0,003 | 0,008 | -0,006 | 0,688 |
| Financial struggles for food | 0,014 | 0,005 | 0,021 | 0,005 | 0,011 | 0,005 | 0,017 | 0,025 | 0,011 | 0,008 | 0,02 | 0,142 | 0,01 | 0,008 | 0,017 | 0,217 |
| Loss of income | 0,066 | 0,017 | 0,027 | <.001 | 0,068 | 0,017 | 0,028 | <.001 | 0,013 | 0,025 | 0,006 | 0,594 | 0,016 | 0,025 | 0,007 | 0,527 |
| Highest obtained degree | -0,018 | 0,008 | -0,015 | 0,017 | -0,016 | 0,008 | -0,013 | 0,045 | -0,012 | 0,011 | -0,012 | 0,269 | -0,012 | 0,011 | -0,012 | 0,287 |
| Employment status | 0,024 | 0,011 | 0,015 | 0,038 | 0,018 | 0,011 | 0,011 | 0,124 | 0,037 | 0,02 | 0,022 | 0,068 | 0,034 | 0,02 | 0,02 | 0,089 |
| Number of cohabiting adults | 0,012 | 0,004 | 0,02 | 0,002 | 0,008 | 0,004 | 0,013 | 0,05 | 0,013 | 0,007 | 0,022 | 0,046 | 0,012 | 0,007 | 0,019 | 0,08 |
| Number of cohabiting children | 0,004 | 0,005 | 0,005 | 0,414 | -0,002 | 0,006 | -0,002 | 0,705 | -0,019 | 0,009 | -0,024 | 0,026 | -0,023 | 0,009 | -0,029 | 0,008 |
|  | *F (11, 241.205) = 16.772, p <.001* | | | | *F (17, 329.201) = 14.845, p <.001* | | | | *F (11, 34.94) = 2.801, p = .001* | | | | *F (17, 56.146) = 2.916, p <.001* | | | |
| *Adjusted R^2^* | 0,006 |  |  |  | 0,009 |  |  |  | 0,002 |  |  |  | 0,003 |  |  |  |
| *Change R^2^* | 0,007 | p <.001 |  |  | 0,002 | p <.001 |  |  | 0,003 | p =.001 |  |  | 0,002 | p = .005 |  |  |
|  |  |  |  |  |  |  |  |  |  |  |  |  |  |  |  |  |
| **Changes in Preparation** |  |  |  |  |  |  |  |  |  |  |  |  |  |  |  |  |
|  | **B** | **SE** | **Beta** | **sig** | **B** | **SE** | **Beta** | **sig** | **B** | **SE** | **Beta** | **sig** | **B** | **SE** | **Beta** | **sig** |
| Constant | 0,251 | 0,047 |  | <.001 | 0,128 | 0,051 |  | 0,012 | 0,19 | 0,073 |  | 0,009 | 0,216 | 0,078 |  | 0,005 |
| *COVID-19 induced feelings* |  |  |  |  |  |  |  |  |  |  |  |  |  |  |  |  |
| Financial Stress | -0,027 | 0,005 | -0,049 | <.001 | -0,027 | 0,005 | -0,049 | <.001 | -0,031 | 0,007 | -0,059 | <.001 | -0,031 | 0,007 | -0,06 | <.001 |
| Feel they have more time | 0,038 | 0,003 | 0,069 | <.001 | 0,038 | 0,003 | 0,069 | <.001 | 0,01 | 0,006 | 0,018 | 0,087 | 0,01 | 0,006 | 0,018 | 0,085 |
| Kessler 6 | -0,013 | 0,005 | -0,017 | 0,011 | -0,013 | 0,005 | -0,017 | 0,013 | 0,017 | 0,009 | 0,022 | 0,062 | 0,016 | 0,009 | 0,022 | 0,071 |
| *Social Distancing Measures* |  |  |  |  |  |  |  |  |  |  |  |  |  |  |  |  |
| Forced to work from home |  |  |  |  | 0,08 | 0,017 | 0,06 | <.001 |  |  |  |  | -0,038 | 0,025 | -0,017 | 0,137 |
| Public gatherings restricted |  |  |  |  | -0,02 | 0,016 | -0,009 | 0,209 |  |  |  |  | 0,001 | 0,024 | 0 | 0,973 |
| Private gatherings restricted |  |  |  |  | 0,015 | 0,02 | 0,006 | 0,44 |  |  |  |  | -0,067 | 0,029 | -0,027 | 0,023 |
| Restaurants closed |  |  |  |  | 0,002 | 0,017 | 0,001 | 0,93 |  |  |  |  | 0,001 | 0,026 | 0 | 0,975 |
| Bars/Pubs closed |  |  |  |  | 0,024 | 0,018 | 0,01 | 0,193 |  |  |  |  | -0,019 | 0,03 | -0,008 | 0,515 |
| Schools closed |  |  |  |  | 0,037 | 0,018 | 0,013 | 0,036 |  |  |  |  | 0,032 | 0,027 | 0,014 | 0,232 |
| *Sociodemographics – control variables* |  |  |  |  |  |  |  |  |  |  |  |  |  |  |  |  |
| Age | -0,001 | 0,001 | -0,021 | 0,005 | -0,002 | 0,001 | -0,023 | 0,003 | -0,001 | 0,001 | -0,015 | 0,23 | -0,001 | 0,001 | -0,013 | 0,305 |
| Financial struggles | 0,004 | 0,004 | 0,008 | 0,326 | 0,004 | 0,004 | 0,007 | 0,367 | -0,005 | 0,007 | -0,01 | 0,477 | -0,004 | 0,007 | -0,008 | 0,55 |
| Financial struggles for food | -0,023 | 0,004 | -0,043 | <.001 | -0,021 | 0,004 | -0,038 | <.001 | -0,008 | 0,007 | -0,016 | 0,225 | -0,008 | 0,007 | -0,016 | 0,218 |
| Loss of income | -0,006 | 0,014 | -0,003 | 0,675 | -0,005 | 0,014 | -0,003 | 0,706 | -0,008 | 0,022 | -0,004 | 0,712 | -0,007 | 0,022 | -0,004 | 0,741 |
| Highest obtained degree | 0,028 | 0,006 | 0,027 | <.001 | 0,024 | 0,006 | 0,024 | <.001 | 0,037 | 0,009 | 0,043 | <.001 | 0,036 | 0,009 | 0,042 | <.001 |
| Employment status | -0,026 | 0,01 | -0,019 | 0,006 | -0,022 | 0,01 | -0,017 | 0,019 | -0,03 | 0,017 | -0,02 | 0,089 | -0,03 | 0,017 | -0,02 | 0,09 |
| Number of cohabiting adults | -0,008 | 0,003 | -0,016 | 0,017 | -0,007 | 0,003 | -0,013 | 0,049 | -0,012 | 0,006 | -0,023 | 0,033 | -0,013 | 0,006 | -0,024 | 0,03 |
| Number of cohabiting children | -0,036 | 0,005 | -0,051 | <.001 | -0,033 | 0,005 | -0,046 | <.001 | -0,044 | 0,008 | -0,062 | <.001 | -0,045 | 0,008 | -0,064 | <.001 |
|  | *F (11, 400.952) = 40.002, p <.001* | | | | *F (17, 441.845) = 28.565, p <.001* | | | | *F (11, 111.938) = 12.009, p <.001* | | | | *F (17, 122.019) = 8.476, p <.001* | | | |
| *Adjusted R^2^* | 0,016 |  |  |  | 0,017 |  |  |  | 0,012 |  |  |  | 0,013 |  |  |  |
| *Change R^2^* | 0,016 | p <.001 |  |  | 0,002 | p <.001 |  |  | 0,014 | p <.001 |  |  | 0,001 | p = .064 |  |  |

a – Separate regressions were used for planning, selecting, and preparing healthier foods for male and female participants. In a first step only personal factors were included, in a second step social distancing measures were added to the model. In both models we controlled for a range of sociodemographic variables known to relate to food literacy. We report the unstandardized beta (B), standard error for the unstandardized beta (SE) and the standardized beta.

b - Sample sizes off all participating countries differed. To control for over or underreporting from certain countries due to unequal survey collections, a survey weight created based on the country proportion in the total sample was applied in all analyses.
